# Design of a Gaze-Controlled Interactive Art System for the Elderly to Enjoy Life

**DOI:** 10.3390/s24165155

**Published:** 2024-08-09

**Authors:** Chao-Ming Wang, Wei-Chih Hsu

**Affiliations:** Department of Digital Media Design, National Yunlin University of Science and Technology, Douliu 640301, Taiwan; popo566464@gmail.com

**Keywords:** gaze estimation, human–machine interface, interactive art, the elderly, active aging

## Abstract

The impact of global population aging on older adults’ health and emotional well-being is examined in this study, emphasizing innovative technological solutions to address their diverse needs. Changes in physical and mental functions due to aging, along with emotional challenges that necessitate attention, are highlighted. Gaze estimation and interactive art are utilized to develop an interactive system tailored for elderly users, where interaction is simplified through eye movements to reduce technological barriers and provide a soothing art experience. By employing multi-sensory stimulation, the system aims to evoke positive emotions and facilitate meaningful activities, promoting active aging. Named “Natural Rhythm through Eyes”, it allows for users to interact with nature-themed environments via eye movements. User feedback via questionnaires and expert interviews was collected during public demonstrations in elderly settings to validate the system’s effectiveness in providing usability, pleasure, and interactive experience for the elderly. Key findings include the following: (1) Enhanced usability of the gaze estimation interface for elderly users. (2) Increased enjoyment and engagement through nature-themed interactive art. (3) Positive influence on active aging through the integration of gaze estimation and interactive art. These findings underscore technology’s potential to enhance well-being and quality of life for older adults navigating aging challenges.

## 1. Introduction

### 1.1. Background

The global aging population is rapidly increasing, with over one billion people aged 65 and older worldwide, a number expected to double by 2050, with up to 40% experiencing disabilities [1]. Aging affects cognitive abilities such as information processing speed, working memory, and long-term memory [2], while physical decline reduces seniors’ quality of life and daily functionality. Active aging, proposed by the WHO in 2002, promotes a healthy, participatory lifestyle for seniors to enhance their well-being.

Advanced technology, including personalized human–computer interaction content, enhances elderly lives and aligns with digital lifestyle trends [3]. Human–machine interaction (HMI) modes, evolving beyond traditional tools like mice and keyboards, now include motion-sensing tech, VR, AR, MR, voice and gesture recognition, and tangible user interfaces (TUIs). Integrating these technologies into elder living spaces fosters health-friendly communication channels and social interactions, promoting inclusivity and improving quality of life [4].

Gaze estimation technology, pivotal in human–computer interfaces, detects real-time eye movements. In applications like fatigue detection in drivers, it analyzes facial features to prevent accidents [5]. In behavioral therapy and VR-based relaxation for seniors, gaze estimation tracks eye movements to tailor activities and potentially aid therapy [6], easing physical interaction burdens and ensuring comfortable, accessible interfaces for elderly users.

### 1.2. Research Motivation

Gerontechnology integrates technology to delay sensory, cognitive, and physical decline in the elderly, emphasizing independence [7]. Elderly users often face challenges with digital technology due to complex interfaces, age-related declines, and lack of prior experience [8], preferring simpler, user-friendly devices.

Today, there are many methods available to mitigate the issues associated with aging in the elderly. Cognitive training enhances older adults’ cognitive abilities and quality of life, promoting independence [9]. As human–computer interfaces diversify, technological integration offers more options for the elderly. Interactive art enriches physical and mental well-being in older adults by integrating new media and music, enhancing seniors’ activity levels and mental health [10,11]. It promotes entertainment, exploration, and social interaction among older adults, fostering a positive mindset [12]. Orange technology combines technological innovation with humanistic care, focusing on health, happiness, and humanitarian needs [13]. Health technology supports seniors’ daily functions through robots like Mini and interactive dialogue systems for social interaction [14,15].

For elderly individuals experiencing physical decline, eye movement remains one of the most active muscle activities. The communication between the user’s gaze and system devices is a noteworthy area of research. Gaze estimation technology aids seniors with physical limitations by facilitating control of smart devices and mobility assistance [5,16]. Eye-based human–computer interfaces are crucial for users with physical mobility limitations, enhancing user experience [17,18].

In summary, interactive art enhances elderly mental health effectively and intuitively. Combining interactive art with gaze estimation technology improves accessibility and usability for seniors, supporting their well-being and independence. In this study, the interaction forms of gaze estimation in interactive art will be analyzed, and an interactive art system that meets the needs of elderly users will be developed. Whether combining gaze estimation with interactive art brings positive effects to the elderly will be verified.

### 1.3. Research Problems and Objectives

The aimed of this study is to examine how interactive art, combined with human–computer interfaces and artistic expression, can offer elderly individuals more novel and meaningful experiential activities. The integration of gaze estimation technology is adopted to broaden the usability of interactive technology products for elderly users with various physical and mental conditions. The following questions will be addressed:

(1) How can the elderly be provided with a smooth and burden-free experience through the design of interactive art experiences with gaze estimation interfaces?
(2) How can the use of interactive art and gaze estimation be developed to promote active aging in the elderly, allowing for them to enjoy meaningful activities in their later life?

The research objectives of this study are as follows:

(1) The analysis of physical and mental conditions and needs of elderly individuals to develop an effective gaze estimation system using highly portable sensing equipment.
(2) The exploration of performance forms of interactive art applications for elderly experiences and the summarization of system design principles.
(3) The development of an interactive art system that integrates gaze estimation with the aim of promoting active aging among the elderly.
(4) The organization of a public exhibition and the evaluation of the system’s usability, enjoyment, and experiential quality through expert interviews and questionnaire surveys.

Whether the above objectives have been achieved will be evaluated in this study by questionnaire survey and expert interviews according to the following indicators:

(1) *User friendliness:* Assessing how effectively a system allows users, particularly older adults, to achieve goals with ease of learning, quick mastery, and thoughtful design elements such as appearance, process simplification, and prompts [19].
(2) *Pleasure perception:* Evaluating users’ responses in interactive contexts, influenced by aesthetic appeal or interactive enjoyment. Positive perceptions can enhance emotional well-being and improve quality of life [11,20,21].
(3) *Interactive experience:* Measuring users’ satisfaction during interactions, encompassing sensory experiences, emotional responses, and social interactions. A high score indicates positive impacts on physical and mental health, stimulating the body, enriching the mind, and slowing cognitive aging [22,23].

### 1.4. Research Scope and Process

**Figure 1:**
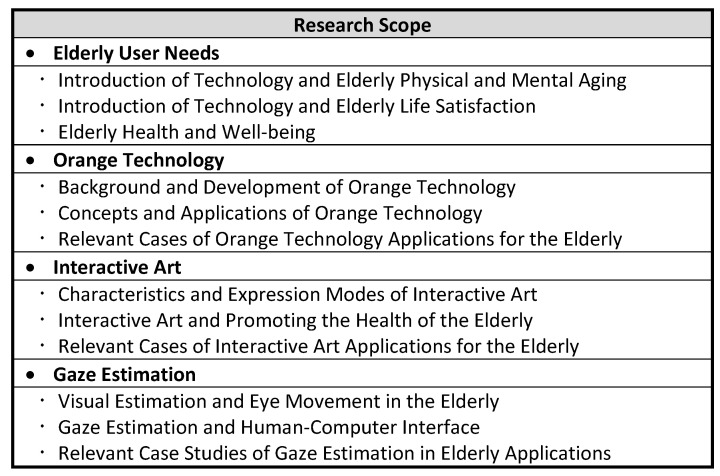
An illustration of the research scope of this study.

The scope of this study includes four aspects: “elderly user needs”, “orange technology”, “interactive art”, and “gaze estimation”, which include the exploration of the current lifestyle of the elderly and the impact of physical and mental aging, as well as the understanding of how to incorporate the technology of gaze estimation into the performance of interactive art. More details of the four aspects are illustrated in Figure 1.

**Figure 2:**
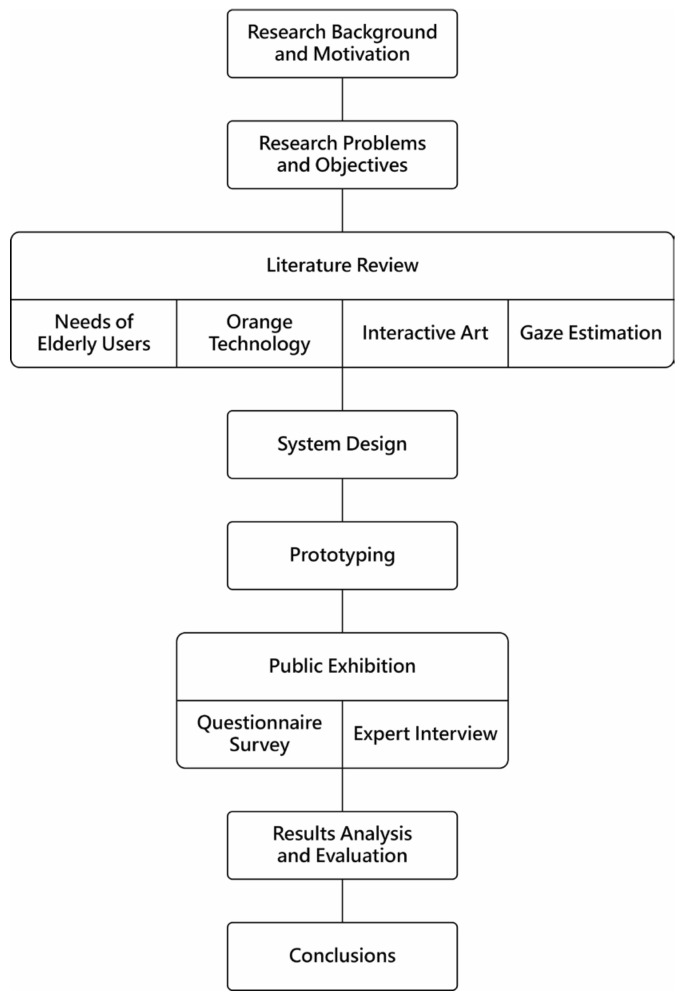
The research process of this study.

The research process in this study was as follows: First, the literature on the abovementioned research scope and some case studies were reviewed to summarize the design principles for the proposed system. After the system’s development was completed accordingly, public exhibitions were held to invite the elderly to participate in experiencing the system. Then, by using questionnaire surveys to collect user experience feedback, combined with results of expert interviews, an analysis and evaluation of the users’ experiences of using the proposed system was conducted. Finally, conclusions were drawn to show the effectiveness of the proposed system. A more detailed research process of this study is illustrated in Figure 2. Implementations of this process are elaborated in the later sections.

### 1.5. Research Limitations

The term “elderly” encompasses a broad spectrum and varies in definition based on age. For the purposes of this study, “elderly” refers to individuals aged 50 and above.

During the experimental process, participants are required to use eye movements to operate and engage with the interactive art system. Therefore, elderly participants must possess basic sensory perception functions. Additionally, this study does not incorporate medically oriented technological applications. Consequently, the research limitations are outlined as follows:

(1) Participants are elderly individuals aged between 50 and 80 years old (inclusive).
(2) Participants must exhibit basic self-awareness and have no cognitive impairments.
(3) Participants must possess basic visual abilities, free from specific eye diseases, and achieve a visual acuity of 0.8 with corrective measures.
(4) The research content and system design do not involve any medical practices.

## 2. Literature Review

In this section, a comprehensive literature review of the four aspects, namely, *the needs of elderly users*, *orange technology*, *interactive art*, and *gaze estimation*, as well as some case studies are conducted.

### 2.1. The Needs of Elderly Users

#### 2.1.1. Introduction of Technology and Elderly Physical and Mental Aging

The acceptance and adoption of technology by the elderly hinge on products’ ability to solve their problems and fit into their lives. Designing for elderly users requires understanding of their changing sensory, cognitive, and motor abilities due to aging [24]. In this study, the aimed is to explore the challenges the elderly face with interactive technology due to physical, cognitive, and psychological aging, aiming to propose design principles for a user-friendly experience.

Visual aging affects acuity, peripheral vision, dark adaptation, color and light sensitivity, and presbyopia [24]. Interface design should prioritize simplicity, clarity, consistency, minimal distractions like flashy elements, and provide clear prompts and feedback [25]. Auditory challenges include hearing loss, especially high-frequency sounds, impacting social interactions [24,25]. Voice interfaces should use low tones and brief feedback messages to reduce cognitive load.

Cognitive decline in reasoning and memory necessitates designs accommodating elderly learning and memory impairments [24,26]. Simple operations, clear feedback, and minimal distractions aid understanding [25]. Complex designs should be avoided, favoring vector graphics and icons over text descriptions to mitigate visual impairments and attention deficits [25,27].

Motor function decline impacts elderly interaction with precise interfaces like mice and keyboards [28]. Design considerations include personalized settings and error-compensating mechanisms like “undo” functions to reduce frustration and enhance motivation [25].

In conclusion, understanding the aging process in the elderly, exploring the challenges they face with technology adoption, and identifying their diverse needs across physical and cognitive domains guide the development of effective design principles. These principles aim to create an experiential environment that meets both the physiological and psychological needs of older adults.

#### 2.1.2. Introduction of Technology and Elderly Life Satisfaction

The quality of life and the physical and mental health of the elderly are closely interlinked. Quality of life is subjectively assessed based on personal perceptions and objectively evaluated against societal standards [29]. Diener [30] defines it as self-assessment and reactions to life conditions, influenced by physical and mental health and overall life satisfaction [31].

Life satisfaction, akin to subjective happiness, reflects individuals’ evaluations of their own lives, integrating emotional responses and past experiences [32,33]. For the elderly, it correlates with psychological health, indicating their attitude toward life and contentment [34,35]. It serves as an indicator for health risks and successful aging [36].

Engaging in meaningful leisure activities is crucial for enhancing life satisfaction, quality of life, and happiness among the elderly. These activities prevent isolation, sustain life purpose, and include self-care, recreation, rest, and social engagement [37]. Integrating technology into their lives further enhances satisfaction and promotes independence [38,39], offering opportunities for entertainment and physical activity.

In summary, the well-being of the elderly hinges on their physical and mental health, life satisfaction, and engagement in meaningful activities. Technology integration enhances their quality of life, fostering independence and happiness. This study focuses on leveraging technology to improve the elderly’s quality of life.

#### 2.1.3. Elderly Health and Well-Being

The WHO’s definition of health from 1948 underscores that health assessment encompasses physical, psychological, and social well-being [40]. Promoting health in older adults requires addressing their physical health, psychological adaptation, and social participation. Global population aging highlights increasing concerns for the physical and mental health of older adults worldwide.

Concepts such as healthy aging, successful aging, active aging, robust aging, and gerotranscendence aim to enhance older adults’ quality of life and maintain their optimism in later years [41]. Healthy aging, introduced by the WHO in 1990, emphasizes lifelong physical and mental health, advocating normal life functions for a fulfilling later life [42]. Successful aging, defined by Rowe and Kahn [43,44], involves avoiding illness, maintaining cognitive and physical function, and remaining engaged in life. Baltes and Baltes [45] expand this concept to include psychological adaptation through selection, optimization, and compensation. Finally, robust aging, proposed by Garfein and Herzog [46], assesses physical function, mental health, cognitive function, and productivity as indicators of resilience, with a focus on social engagement.

Gerotranscendence theory (GTT), by Tornstam [47], encourages older adults to embrace aging positively, fostering wisdom and maturity through self-transcendence on cosmic, self, and social levels. Active aging, introduced by the WHO in 2002 [40], aims to enable older adults to lead healthy, participatory, and secure lives, emphasizing disease prevention, social participation, and personal safety.

The WHO’s “Decade of Healthy Aging 2020–2030” outlines ten priority areas to create age-friendly environments, ensure comprehensive healthcare, and combat age discrimination, aiming for equitable and dignified lives for older adults. Population aging underscores a global imperative, prompting innovative approaches to enhance older adults’ physical and mental well-being. These efforts integrate diverse media to promote peaceful, healthy, and fulfilling lifestyles, offering solutions to challenges associated with aging.

#### 2.1.4. A Summary

The aging-related concepts, objectives, and policies discussed are summarized in Table 1, offering insights into promoting health among the elderly and guiding the development of technology-driven design strategies to address aging-related issues.

Quality of life is a central focus of this study, reflecting the health and happiness of older adults. The reviewed literature underscores that theoretical concepts aim to enhance their quality of life by fostering a positive and optimistic outlook, enabling them to lead healthy, happy, and meaningful lives in later years, albeit from varying perspectives.

In contrast, the new WHO initiative “Decade of Healthy Aging Action” proposes comprehensive guidelines rooted in healthy and active aging principles. It prioritizes the preferences of older adults, advocating for integrated services across families, communities, and policies to promote active aging effectively. Therefore, in this study, the concepts and goals of the WHO project “2020–2030 Decade of Healthy Aging Action” will be followed to further explore the overall picture of the physical and mental development and quality of life of the elderly.

### 2.2. Orange Technology

#### 2.2.1. Background and Development of Orange Technology

Technology advances have significantly enhanced convenience and driven economic growth, improving human quality of life. However, it has also led to societal materialization and environmental deterioration, prompting the rise of green technology focused on environmental protection.

To achieve balanced development between technology and humanity, promoting healthy and happy human life, the concept of orange technology [48] has been introduced. In addition to green technology, orange technology emphasizes human-centered and humanitarian care, aiming to develop technologies and systems for health, happiness, and humanitarian needs [49].

There is a global call for multidisciplinary collaboration among scientists, economists, sociologists, engineers, and other experts to advance orange technology. Initiatives such as Intel’s support for the Center for Aging Services Technologies (CAST) and Harvard University’s CodeBlue project [50] highlight efforts to enhance elderly care through innovative technologies.

Amidst current development trends and theoretical inspirations, while fostering technological advancements, collective efforts are needed to safeguard our environment. Orange technology further underscores the importance of promoting human physical and mental well-being, particularly in designing elder-friendly technological products and creating supportive living environments.

#### 2.2.2. Concepts and Applications of Orange Technology

Orange technology, symbolized by the color orange, representing humanity and humanitarian care, integrates existing information technology and related fields to promote health and happiness. Unlike green technology’s focus on environmental issues, orange embodies the fusion of red’s energy and yellow’s liveliness, advocating for a balanced mind and body.

Rather than developing new technologies, orange technology emphasizes applying, innovating, and integrating existing technologies to create new products aligned with its principles. It actively promotes social care, fundamental values of happiness, and a society where health, happiness, and mutual support thrive.

Through interdisciplinary cooperation, orange technology aims to enhance psychological health and bring warmth and concern to society, fostering an inclusive environment for health and happiness.

(A) *Types of orange computing*

Orange computing encompasses three primary application types:

(1) *Affective computing*: involving capturing emotional cues like tone of voice, facial expressions, and body movements, and including humanizing robots to express emotions.
(2) *Biosignal processing technology*: focusing on gathering vital signs such as blood pressure and heart rate through sensors, aiding healthcare professionals in providing accurate medical advice.
(3) *Advanced companions or assistive technology*: offering advanced companions or assistive technologies that support elderly independence at home, leveraging human-computer interaction, robotics, and sensor devices.
(B) *Types of orange technology*

**Figure 3:**
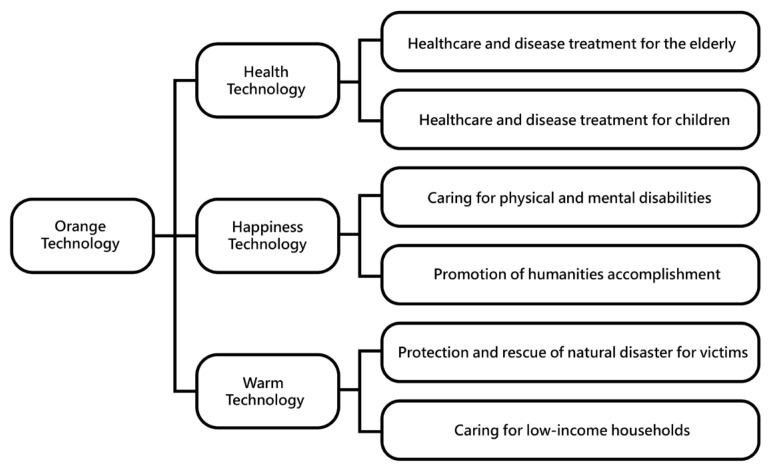
Types of orange technology.

The main conceptual types of orange technology are *health technology*, *happiness technology*, and *warm technology*. Each type can be further divided into specific exploration goals (see Figure 3), including the direction of exploration and promotion objectives [48]. The main conceptual types and their application directions are as follows.

(1) *Health technology*: This includes health and safety care for the elderly and children, disease prevention, and medical diagnosis systems. It also integrates computer communication with medical systems and establishes cloud services for remote medical care.
(2) *Happiness technology*: This involves caring for individuals with physical and mental disabilities, promoting social and humanistic literacy, and measuring happiness indices. Common applications include using biological signals such as blood pressure, heart rate, and exercise frequency to assess happiness, or measuring emotional responses like smiles and laughter.
(3) *Warm technology*: This type focuses on rescue and care for victims of natural disasters and supports socially disadvantaged groups like low-income families. It emphasizes innovative technological applications to enhance human and social interaction, fostering mutual care and community support.

In an era where technology permeates everyday life, orange technology emerges as a novel concept advocating for the development of technology that values human care. It enables socially disadvantaged groups to achieve health, happiness, and social interaction goals through technology, bringing warmth and vitality to people’s lives [48].

Governments, experts, scholars, and organizations worldwide acknowledge the significance of human care, leading to the formulation of diverse policy responses and innovative technologies. Orange technology aligns with this ethos, fostering interdisciplinary collaboration across various fields to evolve and expand existing concepts, thereby enriching the overall framework.

In this study, the concept of orange technology is utilized to develop a system that promotes the health, happiness, social interaction, and overall quality of life for the elderly.

#### 2.2.3. Relevant Cases of Orange Technology Applications for the Elderly

The concept of orange technology aims to bring happiness and joy to humanity with caring as the main concept, making it associated with socially disadvantaged groups, among which are the elderly. In this study, existing cases of orange technology-related products and applications were collected and listed as a reference basis for designing the proposed system.

Nine existing application cases of orange technology, mainly targeting the elderly population, are listed in Table 2 with their types of orange technology, orange computing types, and presentation forms being identified. The type of orange technology is identified according to the three main conceptual types mentioned by Ziyae [48], while the orange computing type is identified based on the technology application trend types summarized by Wang and Chen [49]. The presentation forms are based on the carriers of the presented interactive content.

The analysis and compilation of the cases in Table 2 can be summarized as follows:

(1) Technology advancements enable innovative applications in elderly care, embodying the compassion and warmth of orange technology.
(2) Robots provide diverse services including assistance and companionship, enhancing smart home care for a comfortable and secure living environment.
(3) Interactive devices designed for leisure and entertainment purposes improve hand–eye coordination and mitigate cognitive aging through physical engagement and sensory stimulation.

### 2.3. Interactive Art

#### 2.3.1. Characteristics and Expression Modes of Interactive Art

Artworks historically were unilaterally created by artists to express personal viewpoints through static forms like paintings or sculptures, fostering one-way communication. Advancing techniques now integrate viewers into the artistic process, enabling them to engage with diverse visual presentations, often involving optical illusions, which create a magical art experience. This interaction between viewers and artworks has become central to art, shaping today’s interactive art scene.

Interactive art, propelled by media and technology, transforms traditional artistic expression by offering new media and inspiration sources to artists. Media theorist Manovich [59] identifies its integration across disciplines, highlighting principles such as digital presentation, modularity, automation, variability, and transcoding, distinguishing it from conventional forms. Krueger [60] pioneered “artificial reality” with his “Video Place”, pioneering computer vision-based interactive art, subsequently explored in works like “glowflow”, “metaplay”, and the “video place” series, advancing human–computer interfaces, image interaction, and virtual reality in interactive art creation.

The evolution of the term “interactive art”, transitioning from “digital art” to “computer art” over centuries, now embraces “new media art” [61]. “Media art” underscores technology’s role in enhancing interactive features, drawing parallels with computer games to engage viewers in interactive experiences, fostering curiosity and pleasure as essential components of aesthetic creation.

Interactive art and human–machine interaction (HMI) intertwine, focusing on user experience in artistic creation. HMI explores interactive interfaces and user participation in crafting complete artworks [62]. Virtual reality (VR) exemplifies interactive art, offering unique interactivity and immersive multi-sensory experiences. Interactivity bridges artists and viewers, deepening viewer immersion and understanding of artists’ thoughts and emotions, establishing interactive art as a popular contemporary artistic form [63].

With technological advancements, interactive art explores new media, expanding creators’ ideas. Kelomees [64] studied interactive art systems’ development and applications, focusing on machine vision, computer vision, biological vision, and vision evolution. Their research categorizes interaction modes between interactive art and viewers, captivating viewers with artworks classified into four categories:

(1) *Distance interaction*: Involving cameras or sensors to detect viewers’ behaviors like body movements, eye movements, or sound, triggering feedback events.
(2) *Contact interaction*: Enabling direct physical contact between the system and viewers for tangible interaction and feedback.
(3) *Symbiotic interaction*: Utilizing viewers’ biological signals, such as electro-encephalograms and heart rates, for interaction.
(4) *Chance-based distance and contact interaction*: Incorporating unpredictable viewer behavior into interactive feedback designs, often combining illusions and digital presentations for unexpected effects.

In conclusion, interactive art exhibits characteristics of digitization, multimedia, non-linearity, and interactivity, showcasing the symbiotic relationship between technology and artistic creation. Its value lies in the interaction between artworks and viewers, enhancing user experience and participation levels. This aspect is closely related to human–computer interaction design and extends to applications for the elderly, which is also a significant concern in this study.

#### 2.3.2. Interactive Art and Promoting the Health of the Elderly

Recently, global aging trends have heightened challenges faced by the elderly in terms of both physical and mental health. In response, various solutions and programs have been proposed. Siegler et al. [65] highlighted the evolution of service programs for the elderly in the United States, emphasizing the effectiveness of arts-based practices.

Engaging in artistic activities offers elderly individuals opportunities for self-expression, enabling them to share personal stories and viewpoints. This process supports self-exploration and contributes to emotional well-being, thereby enhancing happiness [66]. Wikstrom [20] discovered that viewing famous paintings promoted feelings of happiness, calmness, and satisfaction among the elderly, correlating with stable blood pressure and improved overall health. Awareness of health status and adaptability also plays a crucial role in leveraging art for physical and mental development [67].

According to continuity theory, artistic engagement helps the elderly connect past experiences with current realities through visual stimuli, fostering satisfaction and social engagement [22]. Moreover, interactive art installations stimulate curiosity and exploratory behaviors among viewers, facilitating community interactions and social engagement [23]. This interactive aspect encourages proactive attitudes and collaborative behaviors, potentially enhancing cognitive functions in the elderly [12].

Human–computer interaction design relies on sensory stimuli to engage users. Visual, auditory, and tactile inputs are integrated into interactive art to enhance viewer immersion [68]. Charitos et al. [69] incorporated environmental experiences into interactive art, promoting participation and communication through sensory stimulation.

Sensory experiences encompass physiological and psychological aspects. Physiologically, sensory stimuli are received by sensory organs, processed by the brain, and transformed into information [39]. Psychologically, sensory experiences involve cognition, emotions, and past experiences. Sensory stimulation positively impacts elderly individuals by enhancing physical activity, cognitive functions, relaxation, and stress relief [11,21].

Interactive art, with its integration of sensory stimulation, holds potential to benefit the physical and mental health of the elderly. Grinde and Patil [70] observed psychological and health benefits for elderly individuals interacting with natural landscapes, including stress reduction. Incorporating animals into interactive experiences enhances acceptance and attention among the elderly, addressing moral and ethical considerations [71]. These insights inform the design of interactive art themes tailored for elderly individuals, aiming to provide enriching and engaging experiences.

#### 2.3.3. Relevant Cases of Interactive Art Applications for the Elderly

Various interactive art forms offer the elderly diverse sensory stimulations. This study showcases examples where interactive art is integrated to promote elderly health, serving as references for designing interactive art systems.

In this study, interactive art applications for the elderly are analyzed based on cases organized into three categories, presentation form, interactive form, and sensory experience, detailed in Table 3. The presentation form categorizes how interactive content is delivered, while the interactive form classifies interaction models based on Kelomees’ proposals [64]. Sensory experience encompasses all elements of sensory stimulation provided to elderly individuals through these interactive artworks.

Elderly individuals demonstrate strong engagement and motivation when experiencing interactive art, which significantly enhances their well-being and quality of life. Interactive technology expands their opportunities to interact with art, bridging generational gaps. This analysis summarizes current trends in the development of interactive art for the elderly, encompassing both domestic and international perspectives.

### 2.4. Gaze Estimation

#### 2.4.1. Visual Estimation and Eye Movement in the Elderly

As the body ages, physical functions such as flexibility, agility, speed, and limb balance decline in elderly individuals, increasing their susceptibility to falls compared to younger age groups [77]. Falls not only impair self-care abilities but also diminish confidence, restrict mobility, and heighten dependence on others, significantly impacting quality of life [78]. Aging also affects visual and sensory capacities, leading to decreased eye movement capabilities [24]. For the elderly, maintaining body posture balance and independent daily living abilities are closely linked to their visual sensory system [79]. Research by Bae underscores that eye movements play a crucial role in posture control for the elderly, who rely on vision to maintain balance [80]. Reduced sensitivity in the soles of the feet further contributes to impaired body balance and an increased risk of falls.

Eye movements serve to optimize visual perception by mapping images onto the retina. They encompass fixations, saccades, and smooth pursuits [81,82]. Fixations involve stable viewing on a target, crucial for receiving visual information. Saccades are rapid eye movements that shift gaze between points in the visual field, categorized into scanning saccades for scene exploration and reactive saccades in response to external stimuli. Smooth pursuit involves tracking a moving object, facilitating smooth monitoring of its trajectory.

Morimoto et al. studied the impact of eye movements and fixation stability on postural balance in healthy adults, highlighting their significant correlation [83]. Addressing visual vestibular system degradation in the elderly through eye movements can activate their vestibular response and improve static balance control [18]. Specifically, saccadic eye movements (SEMs) and pursuit eye movements (PEMs) can enhance plantar skin sensitivity, aiding in posture control and balance during standing [80]. Compared to conventional exercise training, interventions targeting eye movements show greater improvement in lower limb strength and postural balance among the elderly [18].

In conclusion, age-related declines in physical functions, such as reduced foot sensitivity and vestibular sensations, contribute to decreased postural balance and higher fall risks in the elderly. Visual attention plays a crucial role in maintaining balance. Thus, promoting elderly physical and mental health through eye movement interventions supports active aging. This research also explores incorporating eye movements into gaze estimation systems, offering beneficial support for elderly well-being.

#### 2.4.2. Gaze Estimation and Human–Computer Interface

The evolution of human–computer interfaces traditionally involved keyboards and mice, which, while familiar, require precise hand movements and can be cumbersome even with customized setups to simplify operation [84]. Recent advancements in artificial intelligence have ushered in new technologies that enable interaction with computers without heavy reliance on physical movements or portable sensing devices. Techniques like voice recognition, head pose prediction, head-mounted sensors, force sensors, EEG, eye tracking, EMG, and gaze estimation have emerged, with gaze estimation particularly beneficial for users with physical disabilities [85]. As healthcare becomes more critical, human–computer interface considerations are expanding to cater to diverse user needs, including the elderly and individuals with disabilities [19].

Considering the elderly’s declining physical capabilities, eye movements represent one of their more agile muscle areas. Developing user-friendly interfaces using gaze estimation is therefore feasible and can overcome physical limitations, enhance well-being, improve user experience, and boost quality of life [4,17]. Gaze estimation involves analyzing eye features from human face or eye images to determine the focal point or object of interest, facilitating understanding of human intentions through computers or other sensing devices, with deep learning techniques emerging as a pivotal advancement in this area in recent years

**Figure 4:**
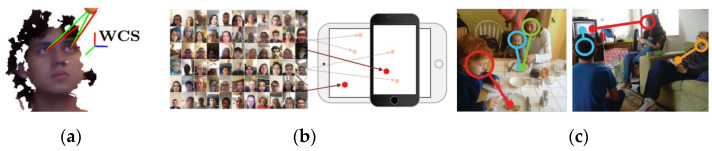
Common task strategies in gaze estimation [86]: (**a**) Three-dimensional gaze prediction. (**b**) Gaze point prediction. (**c**) Gaze target prediction.

Therefore, based on the development of gaze estimation and different scenarios and applications, task strategies can be broadly classified into three types, which are described in the following and illustrated in Figure 4 [86]:

(1) *Three-dimensional gaze prediction*: Predicting eye gaze direction vectors in three-dimensional space based on the relative positions of users and sensing devices.
(2) *Gaze point prediction*: Predicting the focus point of vision, represented in two-dimensional plane coordinates.
(3) *Gaze target prediction*: Predicting specific objects in the input image that a person is looking at.

Common hardware setups for gaze estimation primarily fall into two categories: wearable cameras and remote cameras. Wearable camera methods capture high-resolution images up close but may be uncomfortable for users during interaction due to additional equipment [87]. Remote camera-based methods use fixed cameras at a distance, susceptible to environmental and head motion interference, although deep learning or machine learning techniques can enhance accuracy in such setups [86].

The application of gaze estimation is increasingly active in human–computer interaction and behavior analysis, providing rich insights into human characteristics. This enhances understanding of human behavior and patterns, leveraging eyes as a non-verbal communication technology that fosters social inclusivity through flexible and user-friendly interface operations. This study explores current technological developments in gaze estimation, categorizing them by task strategies and analyzing representative research to guide system development and implementation. The integration of gaze point prediction with interactive art systems aims to enable elderly users to interact stress-free based on eye position, enhancing their engagement and enjoyment.

#### 2.4.3. Relevant Case Studies on Gaze Estimation in Elderly Applications

Eye tracking aids elderly individuals with declining motor functions by enabling computer control through eye movements. In this study, existing cases of applications of gaze estimation in the elderly addressing both mobility impairments and aging-related issues are collected.

The cases of gaze estimation applied to the elderly are listed in Table 4, focusing on sensing devices, technological applications, and application content. Sensing devices are categorized by input methods used in gaze estimation. Based on the analysis from Table 4, the following observations can be drawn:

(1) Compared to traditional human–machine interfaces, gaze estimation enables users to overcome physical limitations with minimal bodily movement, promoting independent living and enhancing quality of life.
(2) Remote sensors are preferred over wearable sensors in device design, maintaining an effective distance to reduce user discomfort during interaction.
(3) Gaze estimation interfaces typically employ computer vision technology, utilizing various types of cameras (depth, infrared, RGB) coupled with deep learning or machine learning to track eye positions.

## 3. Research Methods

**Figure 5:**
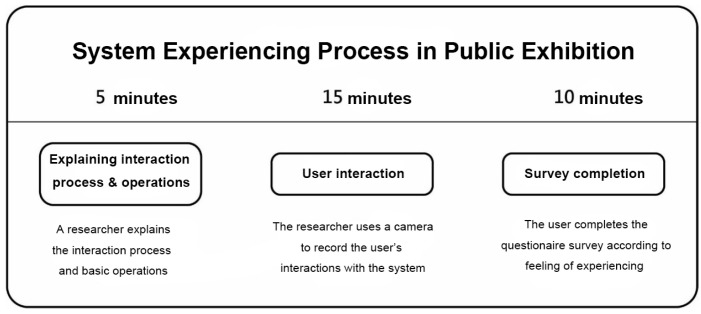
An illustration of the experiencing process of the proposed system in the public exhibition.

The research methods selected for this study include “prototyping”, “expert interviews”, and “questionnaire survey” as the basis for system design and effectiveness evaluation. The system experience process designed in this study is illustrated in Figure 5, and the experiment using the proposed system is as follows:

(1) Experimental site: Elderly learning centers in Changhua, Yunlin, and Chiayi counties and cities.
(2) Subjects: Seniors aged 50 and above, with basic self-awareness and no serious visual impairments.
(3) System experiencing time: Approximately 30 min in total, including 5 min for explanations and operation instructions for the system interaction, 15 min for experiencing the system interaction, and 10 min for questionnaire completion.
(4) Experiencing experiment content: Firstly, a researcher of this study explains the interactive process and basic operation instructions to the participants. After participants have a basic understanding of the overall process, each participating user proceeds with the formal experiencing process while the researcher uses a camera to record the user’s interactions with the system. Afterwards, the participant is invited to complete a questionnaire, with assistance if needed.

### 3.1. Prototyping

Prototyping, in practical system project development, allows for the effective, rapid, and cost-efficient establishment of final user requirements. According to the prototyping process proposed by Naumann and Jenkins [96], it is divided into four main steps: requirement identification, prototype development, implementation and use, and revision and enhancement. The actual situation of system development in this study is adjusted according to this prototyping process, ultimately being organized into the following four processes:

(1) *Requirements analysis*: Through literature review and case analysis, the forms of interactive art expression, potential interaction modes, and usage requirements of the elderly are summarized, while the initial concept of the system is outlined.
(2) *Prototype development*: Based on the initial concept, a system prototype is implemented, with the system’s interactive framework being constructed and the content design conducted, followed by testing the preliminary effectiveness of the prototype.
(3) *Revision* and *improvement*: Interviews are conducted with some target users or experts using the preliminary system prototype, their suggestions to revise the system are referenced, and the details are adjusted until the prototype is perfected.
(4) *System experience*: After the system development is completed, a public demonstration of the system experience is conducted for users to participate in, and user feedback is collected during the process for the final evaluation of system effectiveness.

### 3.2. Expert Interview

The expert interview method is a qualitative research approach aimed at interviewing experts with specific knowledge or recognized social status relevant to the research field in order to explore or collect reliable information [97]. In this study, expert advice was comprehensively collected in a semi-structured manner to obtain high-quality and reliable information.

Expert interviews were conducted during the results analysis and evaluation phase of this study. Three experts in relevant fields were invited for interviews based on the research topic, including cross-domain integrated design, human–computer interaction development, and elderly care and well-being, as shown in Table 5. Each expert interview lasted for 60 min, and numerical identifiers were used as aliases for the experts.

For this study, an interview outline was drafted, as shown in Table 6, focusing on the research topic, and adjustments were made according to the experimental process and interview situations. The discussion primarily revolved around three aspects: “technology integration in elderly activities experience”, “interactive art and active aging for the elderly”, and “application of gaze estimation in interactive art experience”.

### 3.3. Questionnaire Survey

Questionnaire surveys are widely used in social science research to obtain relevant information in a reliable and effective manner, and the accuracy and consistency of the questionnaire are important aspects [98]. Questionnaire surveys can be conducted through face-to-face interviews, telephone interviews, written mails, or by using online forms [99].

In this study, statistical analysis was conducted based on the questionnaire survey results to evaluate the effectiveness of the proposed system. The implementation steps planned for this study are as follows:

(1) Survey timing: After the elderly participants had experienced the system, they were asked to fill out the questionnaire, which took approximately 5 min. We estimated that 50 participants would be surveyed.
(2) Survey method: The questionnaire was distributed anonymously, and it was administered sequentially after the system experience, which took about 10 min. The questionnaires were distributed to the elderly participants before the explanation of the interactive process and operational instructions began.
(3) Steps for conducting the survey: (i) the questionnaire was explained to the participants; (ii) the questionnaire was distributed among the participants; and (iii) the participants were asked to fill out the questionnaire.

This study used the technology acceptance model (TAM), strategic experiential models (SEMs), and the aesthetic emotions scale (AESTHEMOS) for questionnaire design. The questionnaire was adjusted based on the system attributes of this study, and usability and experiential scales for the research system were established. After the participants had operated the system, questionnaires were distributed, and collaborators assisted the interviewees in filling them out. The questionnaires were collected upon completion, and finally, this study analyzed, evaluated, and validated the data from the effective samples.

#### 3.3.1. Technology Acceptance Model

In this study, the *technology acceptance model* (TAM) is utilized to assess the acceptance level of elderly individuals towards a new technology, exploring the application of relevant designs incorporating technology for elderly use. This involves understanding the correlation between the willingness and attitudes of elderly users to design systems that meet their needs. The TAM is widely recognized as a framework for investigating the acceptance of technology products by elderly users [100].

**Figure 6:**
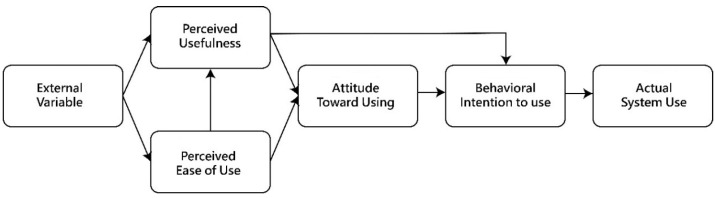
Theoretical framework of the technology acceptance model.

The TAM, proposed by Davis [101], is a theoretical framework designed to predict user behavior and motivation in accepting new information technology. It aims to analyze various factors influencing user acceptance of new information technology and provide reasonable explanations and accurate predictions for user acceptance behavior. The theoretical framework for the TAM is structured as shown in Figure 6.

The TAM framework excludes subjective norm factors and emphasizes perceived usefulness and perceived ease of use as the two primary independent variables influencing technology acceptance. Perceived ease of use indicates that the system is easy for users to operate, requiring minimal effort and time to learn and use the technology. Perceived usefulness indicates that the information system can improve user performance and efficiency, leading to positive user attitudes towards using the technology, which in turn affects behavioral intention to use the technology, ultimately reflected in actual system use. Additionally, perceived ease of use indirectly influences perceived usefulness and, consequently, behavioral intention. Overall, the impact of perceived usefulness on user intention is greater than that of perceived ease of use.

#### 3.3.2. Aesthetic Emotions Scale

To understand the emotional experiences of the elderly towards the system and whether the content can evoke feelings of pleasure, relaxation, or immersion, the *aesthetic emotions scale* (AESTHEMOS) was adopted in this study to assess the emotional responses of the elderly.

The AESTHEMOS, proposed by Schindler et al. [102], is based on aesthetic emotion theories in fields like music, literature, film, painting, advertising, design, and architecture. It provides a framework for measuring aesthetic emotions to evaluate sensory responses and emotional experiences induced by aesthetics. This assessment scale can be used as a tool to examine the aesthetic quality of a work, the effectiveness of interaction with users, and the level of stimulation or impact on users. The scale combines aesthetic experiences from various domains and categorizes them into four emotional expressions:

(1) *Prototypical aesthetic emotions*: Primarily evaluating evaluative aesthetic emotions regarding the style or design of the work, focusing on aesthetic aspects. Expressions used for assessment include “finding it beautiful”, “I like it very much”, “it impresses me”, “deeply moved”, and “very admirable”.
(2) *Epistemic emotions*: Involving emotional experiences of seeking meaning or being touched by the work, delving into deeper levels of observation, covering the novelty or complexity of the work. Expressions used for assessment include “feeling curious”, “sparking my interest”, “challenging”, and “sensing deeper meanings”.
(3) *Pleasing emotions*: Accompanying the experiential feelings during the interaction with the work, including the level of sensory stimulation or finding other meaningful aspects in the work, resulting in pleasant and comfortable emotions. Expressions used for assessment include “making me happy”, “feeling interesting”, “energetic”, “uplifting”, and “feeling relaxed”.
(4) *Negative emotions*: Representing negative feelings without any other implications, although inappropriate aesthetic emotions do exist. Expressions used for assessment include “feeling ugly”, “feeling boring”, “feeling confused”, “feeling repelled”, “feeling anxious”, and “feeling sad”.

#### 3.3.3. Strategic Experiential Models

**Figure 7:**
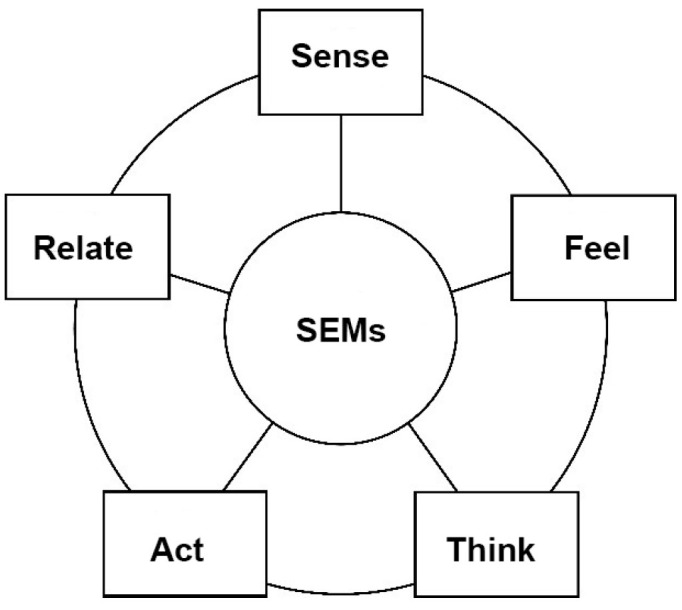
Relationships between the strategic experiential models [103].

In this study, strategic experiential models (SEMs) are employed to assess the elderly’s experiences with the proposed system, aiming to understand their needs and feedback accurately. Developed by Schmitt [103], SEMs aim to create diverse user experiences by analyzing perceptions across five modules: *sense*, *think*, *feel*, *relate*, and *act* (Figure 7). This framework enables a comprehensive evaluation of how the system impacts the elderly’s lives, enriching their spiritual well-being, and promoting physical and mental development. The modules are explained in more detail in the following:

(1) *Sense experience*: Focusing on system-generated sensory stimuli (visual, auditory, gustatory, olfactory, and tactile) to evoke positive user responses.
(2) *Think experience*: Stimulating active problem-solving and creativity, encouraging users to seek information and engage deeply.
(3) *Feel experience*: Aiming to evoke emotional responses and enrich user interaction with positive feelings.
(4) *Relate experience*: Fostering user identification and connection with the system, triggering associations with personal and cultural values.
(5) *Act experience*: Transforming passive engagement into active involvement, prompting users to share experiences, discuss, and take meaningful actions.

These models provide a comprehensive framework for understanding and evaluating user experiences with a system, encompassing sensory, cognitive, emotional, relational, and behavioral aspects.

#### 3.3.4. Design of Questionnaires for Questionnaire Survey

In this study, Likert scale specifications were adopted for questionnaire design. The scale categorizes question attributes into five response options: “strongly disagree”, “disagree”, “neutral”, “agree”, and “strongly agree”, each assigned a numerical score of 1, 2, 3, 4, or 5, respectively. Following the TAM proposed by Davis [101], the questionnaire was designed to assess the operational experiences of the elderly regarding the implementation of gaze estimation interactive interfaces. Participants were asked to fill out the questionnaire to evaluate the impact and usability of incorporating gaze estimation on the elderly.

Furthermore, based on the SEMs introduced by Schmitt [103] and the AESTHEMOS proposed by Schindler et al. [102], a survey was conducted to investigate users’ experiential feelings with interactive art systems. This evaluation aimed to assess the effects of interactive art experiences on the physical and mental development of the elderly, validating the system’s pleasantness and experiential quality. The questionnaire was divided into three scales: “user friendliness”, “user satisfaction”, and “user experience”. To ensure accessibility for the elderly, questionnaire items were designed in a straightforward and colloquial manner. The descriptions of the three scales are as follows:

(A) *Questionnaire design for the scale of user friendliness*

The main goal is to evaluate if integrating gaze estimation into tech applications for the elderly is suitable, assessing their acceptance and usability. The user friendliness questionnaire is based on Davis’s TAM [101], tailored for this study to create the scale of “user friendliness assessment”. It includes 10 questions, detailed in Table 7.

(B) *Questionnaire design for the scale of user satisfaction*

The aim is to determine if older adults experience positive emotions using the interactive art system and to evaluate their perceptions of the experience and thematic environment design. This assesses the pleasant feelings users have while using the system. The assessment of pleasant feelings in this study is primarily based on the AESTHEMOS framework proposed by Schindler et al., adapted to fit the theme of this research, resulting in the creation of the scale of “user satisfaction assessment”. The questionnaire comprises 10 questions, detailed in Table 8.

(C) *Questionnaire design for the scale of user experience*

The main goal is to assess if older adults find the system design satisfactory and whether it enhances their lives, potentially influencing their daily behaviors and improving their overall quality of life. The assessment of interactive experience in this study is based on Schmitt’s strategic experiential models (SEMs), adapted to fit this study’s theme, resulting in the creation of the scale of “user experience assessment”. The questionnaire is detailed in Table 9.

## 4. Design of Proposed System

Introducing technology into elderly care creates more meaningful activities, integrating advancements increasingly into daily life. Professionals advocate for applying technology across fields to enhance elderly physical and mental well-being, aiming for a comfortable life. Interactive art applications particularly benefit elderly well-being by stimulating senses, promoting psychological activation and motivation [4].

Elderly individuals often experience reduced physical agility but maintain good eye control, facilitating the development of intuitive human–machine interfaces. Gaze estimation serves as an interactive interface, particularly in interactive art systems, as adopted in this study. This approach aims to provide enjoyable sensory experiences for the elderly, integrating key eye movement events to encourage active aging.

### 4.1. Design Concept

Based on Grinde and Patil’s [70] findings, interactions with nature enhance the well-being of older adults through meaningful activities. Valk et al. [71] suggest that integrating animal ecology further captures their attention. This study introduces “Natural Rhythm through Eyes”, integrating gaze estimation technology into interactive art experiences tailored for older adults. This system uses gaze fixation, scanning, saccade response, and smooth pursuit—four eye movement events—to engage users with thematic interactive content, aiming to trigger varied system responses.

These interactive experiences are expected to enhance older adults’ technological engagement and understanding. The system aims to provide relaxing, stress-relieving experiences with sensory stimulation for comfort. Moreover, by incorporating eye movement events, it aims to reduce operational burden while enhancing physical functionality to delay cognitive decline and promote physical activation. Through these interventions, this study aims to support active aging and cultivate a positive outlook on late-life experiences amid natural aging processes.

### 4.2. System Design

In this study, the TensorFlow Lite (TFLite) and Open Source Computer Vision Library (OpenCV) frameworks are employed to develop a gaze estimation model. By analyzing facial images of users, the model estimates the user’s gaze position on the screen, which serves as the basis for developing a human–computer interface for an interactive art system tailored for older adults.

The system features eight distinct natural environments: deep sea, shallow sea, desert, starry sky, garden, forest, rainforest, and clouds. Each environment offers unique visual scenarios, designed to provide older adults with a hierarchical and fantastical experience based on natural landscapes. The overall system setup includes a network camera as an input sensor, and a display screen with audio speakers for media output.

The development of the system in this study is divided into two main parts:

(1) Gaze estimation system design: focusing on developing an interactive interface based on gaze estimation.
(2) Interactive art system design: focusing on presenting interactive content based on the identified gaze position.

These components work together to create a rich interactive experience that combines technological innovation with natural-themed visual and auditory stimuli tailored to enhance engagement and well-being among older adults.

The gaze estimation system in “Natural Rhythm through Eyes” functions as the primary interface, using a webcam to capture facial images and estimate the user’s gaze position on-screen in pixels. This allows for users to control heat map points and interact with scene objects. Compared to wearable sensors, using a remote camera reduces discomfort for elderly users, with effective operation up to approximately 60 cm. Interaction via eye movements is intuitive, enabling users to adjust their gaze on-screen to control processes, enhancing usability. The system promotes active aging by integrating eye movements—fixation, saccades, smooth pursuits, and reaction saccades—into each interactive phase, encouraging engagement.

The interactive art system “Nature’s Gaze” relies on gaze estimation for dynamic visual presentation and interactive communication. Upon detecting users’ facial features, the system initiates experiences instantly. Each environment features distinct natural landscapes like deep sea, shallow sea, desert, starry sky, garden, forest, rainforest, and cloudscape designs. Operational modes correspond to different eye movements such as fixation, saccades, reaction saccades, and smooth pursuits, facilitating interaction with thematic scenes throughout various phases.

To immerse elderly users in natural settings, “Natural Rhythm through Eyes” presents varied natural scenery at each interaction stage. These scenes feature dynamic visual effects and soothing background music, enhancing relaxation through visual and auditory sensory experiences. Elderly users engage with and explore these natural landscapes using eye movements. Table 10 illustrates the dynamic visual designs of the scenes presented in “Natural Rhythm through Eyes” within the interactive art system.

### 4.3. System Process

The interactive art system experience design is divided into six stages, each describing the visual scene design and the objectives for that stage as described below:

(1) Stage 1: Visual design set in the scene of Deep sea, where the feedback camera captures the screen continuously until the user is near the center of the camera. This stage primarily adjusts and confirms the initial position of the user’s head.
(2) Stage 2: Visual design set in the scene of Shallow sea, featuring a regular animation of fish movements to attract user attention. It guides the user’s gaze to track the fish’s position, while the system captures gaze data to achieve gaze calibration.
(3) Stage 3: Visual design set in the scene of Desert, with randomly appearing dots of light to stimulate user response. Users are encouraged to fix their gaze on the light dots, triggering their disappearance. The number of triggers confirms the effectiveness of the calibration and the user’s intuitive control.
(4) Stage 4: Visual design set in the scene of Starry sky, where three themed and one random preview images are evenly distributed on the screen. Users can choose a theme to experience by gazing at their preferred option, and all choices can be repeated.
(5) Stage 5: Entry into the selected theme experience. After experiencing the chosen theme, users obtain a theme experience result based on their interactive operations. The four theme choices are described as follows:
  (i) Star Garden: The scene Garden, themed with flower species, interacting with flowers through scanning saccades and gaze eye movements.
  (ii) Animal Forest: The scene Forest, themed with animal species, interacting with animals through gaze eye movements.
  (iii) Forest Trees: The scene Rainforest, themed with tree species, interacting with trees through scanning saccades and gaze eye movements.
  (iv) Random Theme: Choosing this option randomly enters Star Garden, Animal Forest, or Forest Trees for the experience.
(6) Stage 6: Visual design set in the Cloud layer scene. After the user completes experiences in three themes, the system generates a correlated natural landscape drawing based on the user’s interactions with the three themes, providing the final experience result.

In Stage 5, each theme experience has four representative species categories. After completing a single theme experience, the system determines the user’s primary focus species based on their gaze time for each of the four species in that theme. The final theme result provides users with the symbolic meaning of that species. The representative species and their symbolic meanings for each theme are detailed in Table 11.

The interactive system “Natural Rhythm through Eyes” developed in this study targets individuals aged 50 and above. Considering the aging process of this demographic, the operation is designed to be simple, advocating for a relaxed experiencing process. This allows for users to immerse themselves and enjoy the sensory experiences brought by interactive art. The experiencing process integrates feedback based on eye movement exploration of scenes, enabling elderly users to comfortably engage throughout.

**Figure 8:**
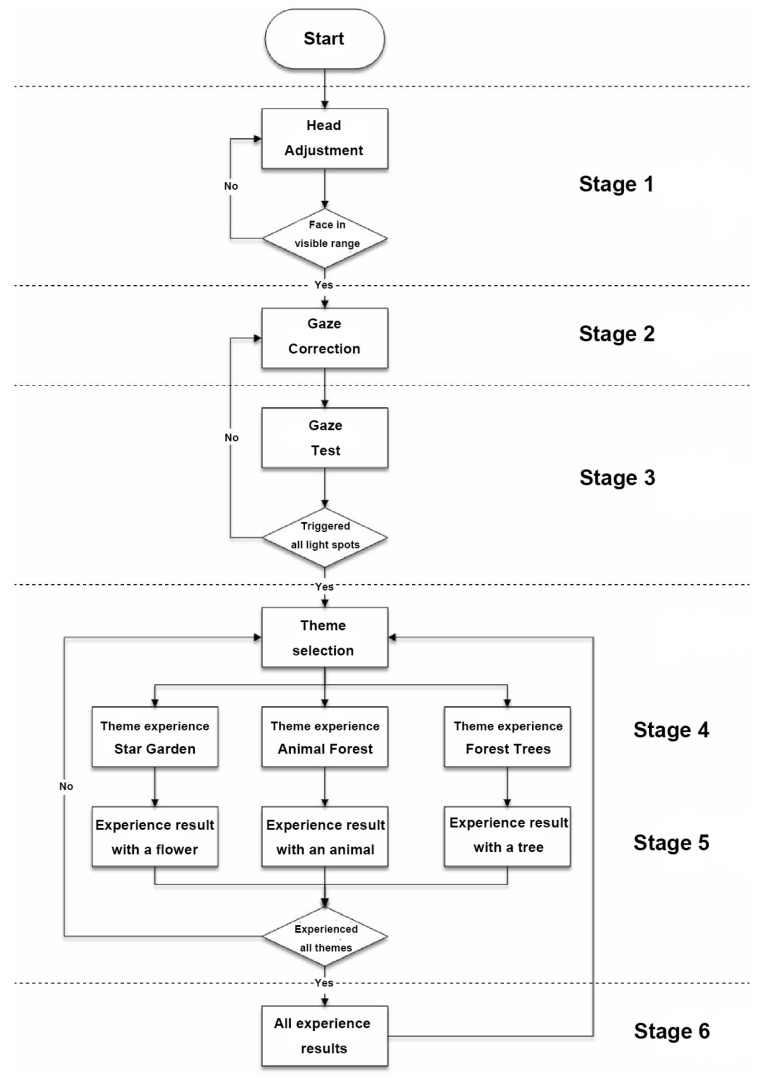
Flowchart of the proposed system “Natural Rhythm through Eyes”.

**Figure 9:**
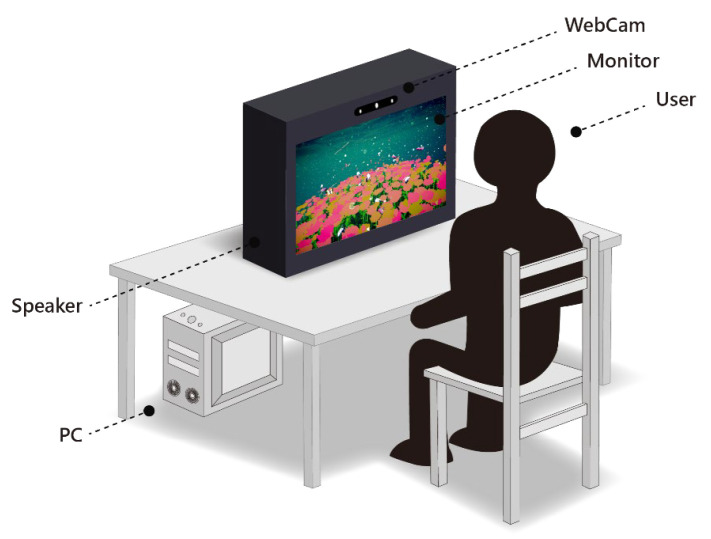
The interactive situation of “Natural Rhythm through Eyes”.

The entire process follows a coherent sequence of natural-themed scene transitions, with only one decision event, while the rest is system-guided, minimizing user operational demands. The system flow of “Natural Rhythm through Eyes” is depicted in Figure 8, and the detailed explanations of each interaction stage are provided subsequently. A series of illustrations to show the detailed steps of the system process is shown in Table 12, with each step of operating the system “Natural Rhythm through Eyes” explained in detail by text in the rightmost column of the table. The interactive situation of “Natural Rhythm through Eyes” is shown in Figure 9.

**Figure 10:**
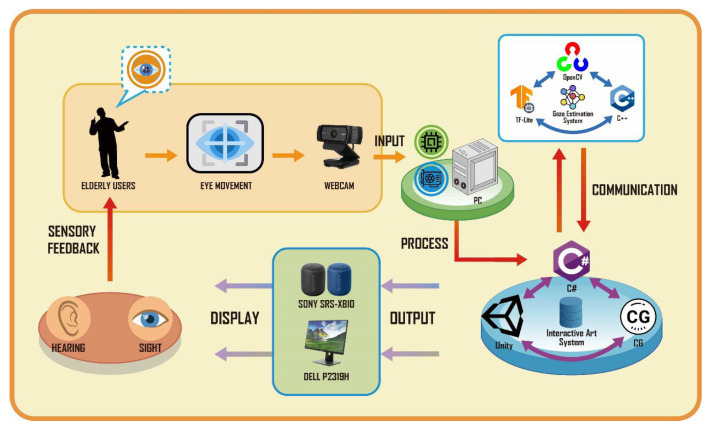
The architecture of the proposed system “Natural Rhythm through Eyes”.

The system architecture of the interactive system “Natural Rhythm through Eyes”, developed in this study, is illustrated in Figure 10. Below is an explanation of each component in sequential order, forming a cyclical structure within the system:

(1) *System input*: Users interact with the system through eye movements (Eye Movement). The system captures user facial images using a webcam (WebCam) as the input source for the interactive system. These inputs are integrated by the interactive art system and further processed to interpret user interaction behaviors.
(2) *Gaze estimation system*: Built using C++, integrating TensorFlow Lite (TFLite) and the OpenCV open-source library to construct deep learning and machine learning models. It analyzes user facial images received from the interactive art system, extracts image features, and estimates the user’s gaze coordinates on the display screen.
(3) *Interactive art system*: Constructed using Unity for creating interactive art experience environments. Front-end control of the interactive system was programmed in C#, and visual effects were developed using CG scripting. This system also integrates the gaze estimation system, continuously receiving estimated gaze coordinates. These coordinates are used for real-time front-end interaction control and feedback for visual performance.
(4) *System output*: Visual images and audio effects from the interactive art system are outputted through the monitor and speakers, providing users with sensory feedback (Sensory Feedback) in terms of visual and auditory experiences.

This cyclical system architecture ensures seamless interaction between the user’s gaze input, processing through the estimation system, integration into the interactive art environment, and final sensory output to the user.

### 4.4. System Development

**Figure 11:**
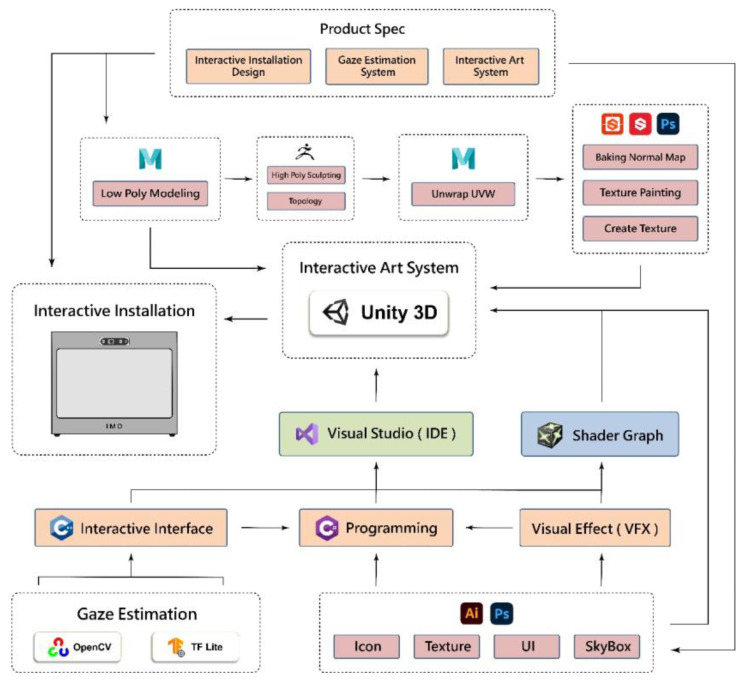
The development flow of the proposed “Natural Rhythm through Eyes” system.

As illustrated in Figure 11, the proposed system “Natural Rhythm through Eyes” includes two separate major parts: the gaze estimation system and the interactive art system, accompanied by an interactive installation design (see the top block in the figure). The overall development process includes (a) the creation of 3D and 2D scenes and the design of object design in the artistic aspect, and (b) the implementations of the front-end controls, algorithms, and scene shaders in the system development aspect. The programming languages, frameworks, development software, and algorithmic processes used in the system are described in detail in the following:

(A) *In the artistic aspect*

For 3D artistic scene construction, Maya software (version 2020) was used for modeling, animation binding, and UV unwrapping. Further sculpting was carried out using ZBrush, and textures were ultimately created using Substance Painter, Substance Designer, and Photoshop. For 2D artistic scenes, Photoshop and Illustrator were utilized to design materials for the UI interface, which included special effects textures integrated with shaders. Visual presentations in some scenes not only relied on static material setups but also incorporated shader effects programming. Shader programming was accomplished using Shader Graph (version 14.0.10) and VFX Graph (version 14.0.10) within the Unity engine (version 2022.3.20).

(B) *In the system development aspect*

The interactive art system’s control flow was integrated using Unity and written in the C# language. The gaze estimation system utilizes C++ combined with image processing through OpenCV and the neural network framework TFLite. After completion, it was compiled into a DLL and connected to the Unity end.

#### 4.4.1. Gaze Estimation System Process

**Figure 12:**
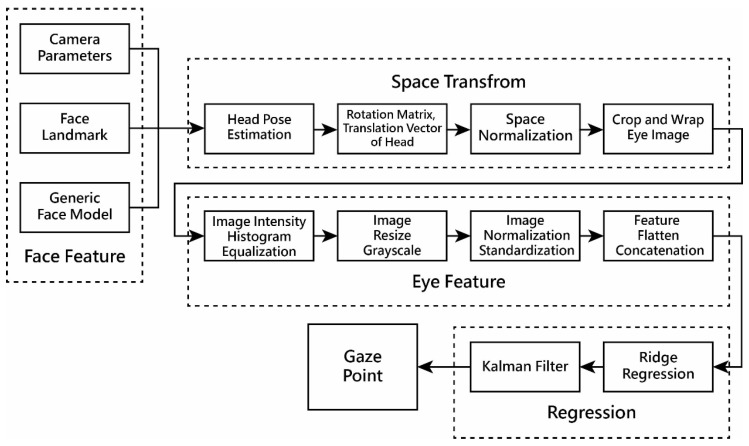
The gaze estimation process of the proposed “Natural Rhythm through Eyes” system.

With an RGB camera for image acquisition, a gaze estimation method is designed in this study, using the eye image as the input, to estimate the gaze-point position in terms of screen coordinates. The process is depicted in Figure 12 and described in the following:

(A) *Face feature extraction stage*

An initial stable development framework, including the processes of face detection, face landmark detection, and iris landmark detection, was initially employed to locate comprehensive facial information and extract various face features from the input image acquired by use of a single-eye RGB web camera.

(B) *Space transform stage*

To compensate for *user head rotations, tilting, and movements* during camera use, the input image undergoes transformation into a unified space. This involves *head pose estimation* using data from a static 3D face model, real-time-detected *facial and iris landmarks*, and camera parameters. The fitted *head pose information*, including the *rotation matrix and translation vector*, is then normalized to standardize the spatial capture of *user facial images* from various angles. A *perspective matrix* is subsequently derived to transform the image of the *user’s eyes* in the standardized position, resulting in a *cropped and wrapped eye image*.

(C) *Eye feature extraction stage*

After obtaining the local eye image that includes the left and right eyes, an image preprocessing process is conducted, including the steps of image *intensity histogram equalization*, *image resizing*, and *conversion to grayscale*, followed by *image normalization* and *standardization*. Then, the eye features from both eyes are *flattened and concatenated* to obtain a dual-eye feature vector for the next stage.

(D) *Regression stage*

Finally, a *ridge regression* model is utilized to learn the mapping from the eye features of the standardized head pose to the coordinates of the gaze point. During this stage, the estimated values are further smoothed using a *Kalman filter* to reduce noise, resulting in the stable final gaze point as the output.

#### 4.4.2. Interactive Art System Process

**Figure 13:**
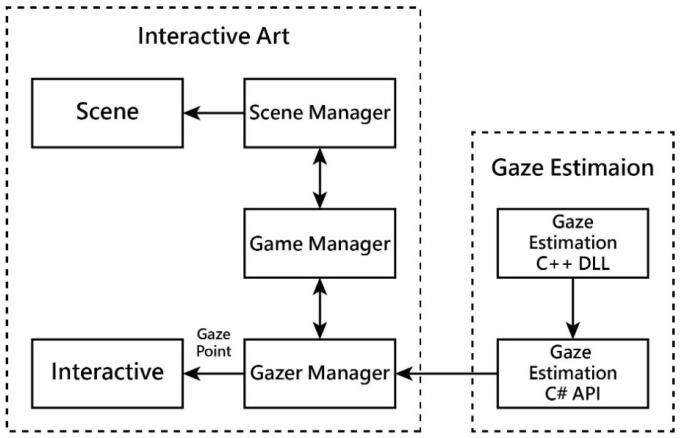
An illustration of the interactive art system’s process (the details of the gaze estimation process are shown in Figure 12).

We integrated the interactive art system with the gaze estimation system using the Unity3D game engine to create the proposed “Natural Rhythm through Eyes” system. This interactive art system follows a six-stage process, previously illustrated in Table 12 and depicted in Figure 8, where each scene generated in the six stages involves different contexts and themes managed by respective modules, as shown in Figure 13. Simultaneously, the gaze estimation system operates persistently in the background of the interactive art system, providing real-time estimation results upon system activation.

More specifically, as shown in Figure 13, the interactive art system is managed by three modules: *scene manager*, *global game manager*, and *gazer manager*. The global game manager and the gazer manager operate in the background, while the scene manager adapts its module configuration based on different thematic scenes. The following are the detailed explanations of the three manager modules:

(1) *Gazer manager*: developed in C++ to manage gaze estimation, compiled into a DLL, and wrapped with an interface written in C# within Unity3D, allowing for it to be called as an API and executed according to Unity’s operational standards.
(2) *Game manager*: a central management tool written in Unity’s scripting language that controls the overall system state and processes, including scene initialization and interactions between modules.
(3) *Scene manager*: initializing scenes based on their themes; managing object generation, scene effects, user interface (UI) display, and transitions/loading between scenes; and handling of relatively static scene object controls.

These modules collectively enable the interactive art system to function smoothly within Unity3D, as depicted in Figure 13. Within the main three managers of the interactive art system, they can be further categorized into two modules to distinguish between types of sensory feedback in interactive art: *static sensory feedback scene module* and *dynamic interactive feedback module*.

(1) *Static sensory feedback scene module*: Controlled by the scene manager, this module handles the initialization of themed scenes. It includes setting background music and sound effects specific to the theme, initializing scene objects, adjusting parameters, and displaying dynamic background visuals. This module focuses on static visual and auditory feedback.
(2) *Dynamic interactive feedback module*: This module utilizes the output of gaze estimation, particularly the gaze point, to interact with objects within the interactive art system. It manages effects generation, object manipulation, model animations, display of gaze-point heatmaps, and other dynamic interactions within the system.

These two modules provide users with different types of sensory feedback interactions, enhancing their overall experience within the interactive art system.

#### 4.4.3. Major Steps in implementing the Gaze Estimation and Interactive Art Processes

**Figure 14:**
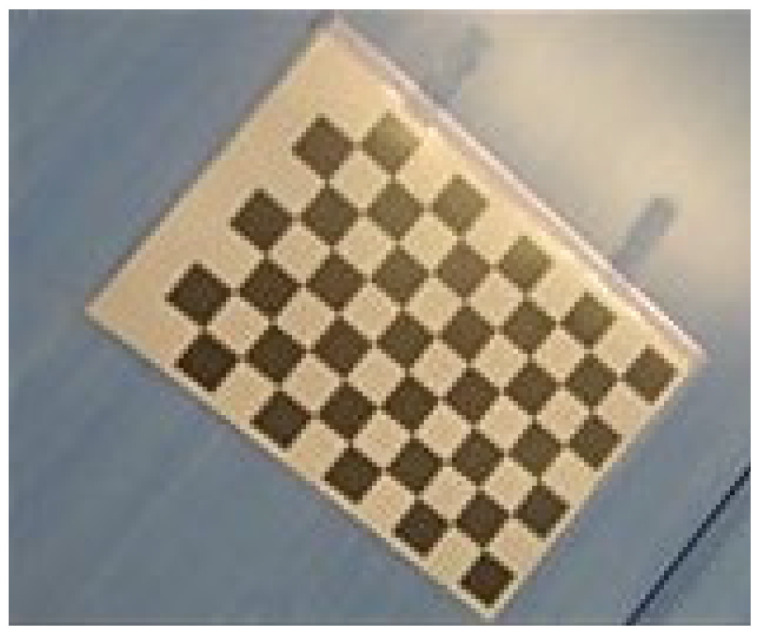
A checkerboard used for camera calibration in this study.

**Figure 15:**
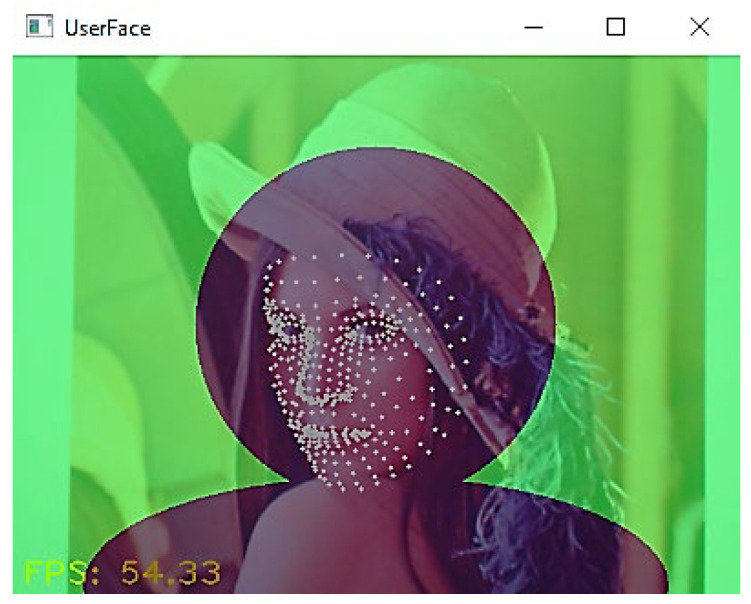
An example of face landmark detection results by the MediaPipe framework.

The implementations of some major steps conducted in the processes of the gaze estimation and interactive art systems are explained here. Refer to Figure 14 and Figure 15 when necessary for the discussions in the following.

(A) *Camera calibration*

The initial step in gaze estimation involves extracting facial features. The first task is calibrating the camera to acquire its intrinsic and distortion parameters, *C*_c_ and *D*_c_, respectively. A checkerboard pattern, depicted in Figure 14, is utilized to capture 30 images from different angles. These images are then processed using the *calibrateCamera*() procedure in the OpenCV library to obtain the parameters *C*_c_ and *D*_c_, which are crucial for subsequent stages of gaze estimation.

(B) *Face and iris landmark detection*

In this study, the detection of face landmarks is carried out by utilizing Google’s MediaPipe framework [104], which incorporates a face landmark detection module and an iris landmark detection module, both based on deep learning. Figure 15 illustrates a sample of the test results from the face landmark module, where the white dots indicate detected facial landmark points in the input image “Lina”. In total, 478 feature points are detected by these modules, serving as the input data for the gaze estimation process.

(C) *Head pose estimation*

**Figure 16:**
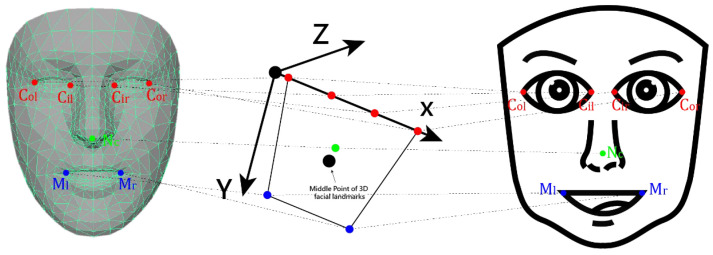
An illustration of the concept of user head pose estimation with the left part showing a generic face model and the right part showing a face composed of the facial and iris feature points extracted from the user’s input face image.

The process of head pose estimation in this study utilizes a generic face model, the set of 478 feature points extracted from the face image mentioned previously, and the calibrated camera parameters *C*_c_ and *D*_c_. The generic face model required for this purpose is sourced from the MediaPipe framework, as shown in the leftmost part of Figure 16. The rightmost part of the figure illustrates the representation of the face using the 478 extracted face feature points.

To estimate the head pose represented by the 3D *head coordinate system* X-Y-Z in the middle part of Figure 16, the set of the *seven main facial reference points* in the left face model (namely, the left eye inner corner *C*_il_, left eye outer corner *C*_ol_, right eye inner corner *C*_ir_, right eye outer corner *C*_or_, nose center *N*_c_, left mouth corner *M*_l_, and right mouth corner *M*_r_) and the set of those corresponding reference points in the right face are taken out to form seven corresponding point pairs. These point pair data are taken as the input to the Levenberg–Marquardt algorithm in OpenCV software to yield the output of a rotation matrix *R*_w_ and a translation vector *t*_w_, which are then taken to represent the head pose of the user (equivalent to the rotation and translation from the world coordinate system to the camera coordinate system).

(D) *Space transformation*

The step of image space transformation ensures that the eye image captured by the camera can be uniformly transformed, via a perspective matrix, to a standardized appearance in the calibration space. This standardized appearance image represents how the camera should capture the user’s face when it faces the face directly from a perpendicular direction. This method of obtaining the standardized appearance image helps mitigate the effects of head movement to some extent.

**Figure 17:**
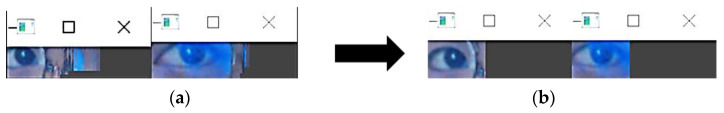
An example of the results of perspective space transformation applied to the two eye images: (**a**) Before the transformation. (**b**) After the transformation.

The process of the previously mentioned perspective space transformation is carried out by performing the function *warpPerspective*() in OpenCV for the partial images of both the left and right eyes. An example of the results is shown in Figure 17.

(E) *Extraction of eye features*

**Figure 18:**
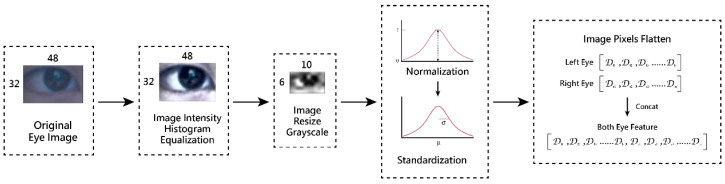
An illustration of the eye feature extraction process.

After obtaining the appearance images of the eyes through the space transformation, a sequence of image preprocessing steps is conducted initially. The first is to perform histogram equalization on the image intensity to balance the brightness and contrast, thereby reducing the influence of natural lighting conditions. This process effectively enhances the contrast and brightness distribution, thereby improving the visual quality of the images. Then, the images are converted to grayscale ones, and normalized and standardized to be of the size of 10 × 6 pixels. The image pixels are then flattened and concatenated to obtain a 1 × 120 feature vector. The abovementioned operations of eye feature extraction are illustrated in Figure 18.

(F) *Use of ridge regression and Karman filtering to enhance gaze accuracy*

To enhance the accuracy of the estimated gaze-point position, this study adopts the ridge regression technique, depicted in the bottom-right corner of Figure 12. The regularization method incorporated in the ridge regression model mitigates multicollinearity to some extent, allowing for more precise estimation of the gaze-point location with a smaller number of input samples. This approach helps address issues that arise when the number of features exceeds the number of samples.

**Figure 19:**
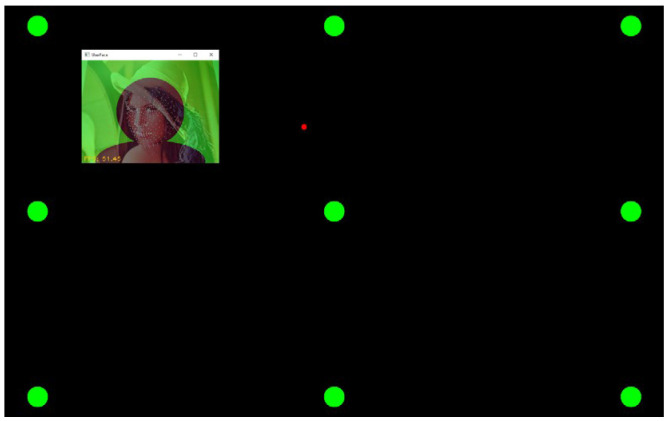
Gaze estimation by use of 3 × 3 grids of green calibration points on a monitor.

In this study, to learn the parameters of the regression model that maps the extracted eye features to the gaze-point coordinates, sample eye images used as input to the regression model are manually created. This is achieved by mouse clicking on the full screen of a monitor marked with 3 × 3 grids of calibration points (the green dots), as shown in Figure 19.

**Figure 20:**
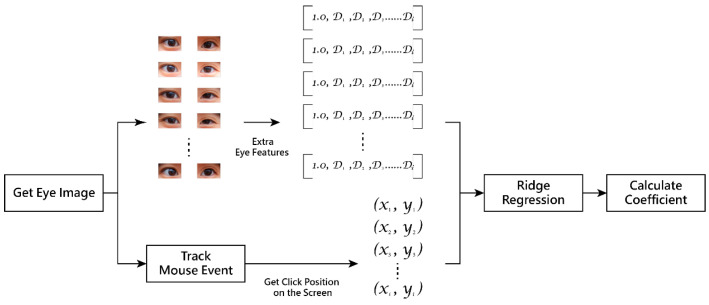
An illustration of the learning process of ridge regression for gaze estimation.

Assuming the user’s gaze position corresponds to the current position of the mouse cursor, whenever a click event is triggered on the screen, the aforementioned image preprocessing steps are applied to the current facial image to extract the 120 features of the two eyes, as described earlier. This process is repeated to generate a series of at least 30 samples, each consisting of a 1 × 120 feature vector along with the mouse click coordinates (*x_i_*, *y_i_*). These samples form the input dataset for training the regression model, as illustrated in Figure 20.

Furthermore, after the feature data undergo ridge regression to determine the gaze point position, any potential noise in the resulting gaze point data is filtered using the Kalman filtering technique. This technique predicts the gaze-point position based on sequential gaze measurements over time, integrating both current and past measurements to enhance estimation accuracy, as depicted in Figure 12.

(G) *Interactive interfacing triggered by collision of the gaze vector and the object*

**Figure 21:**
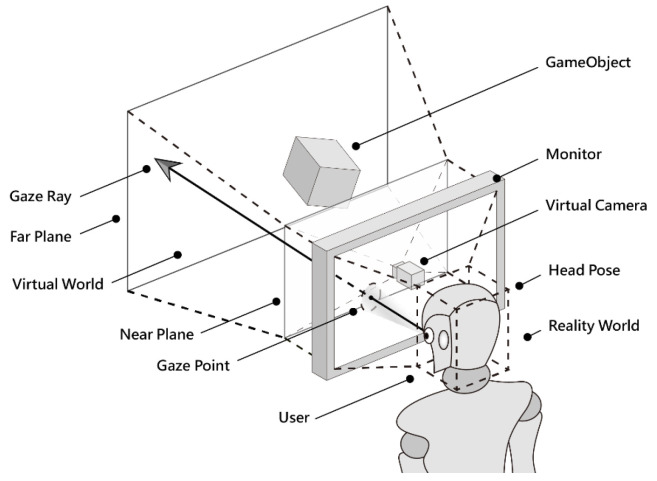
An illustration of the interactive interface of the proposed system.

The proposed interactive art system continuously integrates the gaze-point estimation result into the virtual space created using Unity software. This transformation into world coordinates enables the system to construct a vector ray that represents the user’s gaze direction in space. The system then determines whether the user’s current gaze direction intersects with interactive objects within the virtual environment. Upon collision, interactive feedback corresponding to the current theme, outlined in Table 12, is generated. Figure 21 illustrates the conceptual interface of the proposed interactive art system, showcasing an example with a perspective camera.

**Figure 22:**
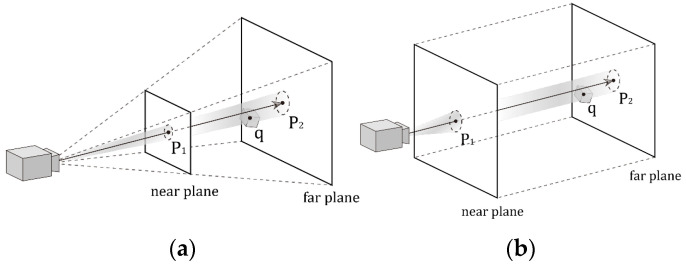
An illustration of deciding whether the center point of an object is contained within the expanded volume range of the camera ray’s direction: (**a**) Case *a*: using a perspective camera. (**b**) Case *b*: using an orthographic camera.

More specifically, the process to decide whether the user’s gaze direction collides with an object in the virtual space can be decomposed into the following detailed steps:

(1) Transform the screen coordinates into the world coordinate system of the virtual space.
(2) Determine whether the center point *P*_c_ of an object is contained within the expanded volume range of the camera ray’s direction. There are two ways to make this decision, as described in the following:
  (a) *Under the assumption of using a perspective camera*: As illustrated in Figure 22a, in this case, the goal becomes to decide whether the point *P*_c_ appears within the *cone* shape formed by the gaze vector as the central axis.
  (b) *Under the assumption of using an orthographic camera*: As illustrated in Figure 22b, in this case, the goal becomes to decide whether the point *P*_c_ appears within the *cylinder* shape formed by the gaze vector as the central axis.

(H) *Design of system scenes in the process of the interactive art system*

The interactive process of the system comprises six stages for user experience, and includes a total of ten scenes for use in the stages, as depicted in Table 10 and Table 12, respectively. The contents of the scenes and dynamic objects (flowers, animals, trees, etc.) in them, like those in Table 11, are designed by use of the graphic tools shown in Figure 11. Furthermore, during the loading process of each scene, fade-in and fade-out effects are implemented to mitigate potential visual stuttering caused by scene transitions.

The most important is the interaction of the scenes and objects with the user, which is based on the user’s control of his/her gaze point on the screen. The estimation of the gaze-point position is carried out by the proposed gaze estimation system, as described previously in this section.

## 5. Research Results

This research was conducted with five public demonstrations of the proposed system “Natural Rhythm through Eyes”, which combines interactive art with gaze estimation, all being held in senior learning centers in Changhua, Yunlin, and Chiayi counties in Taiwan in March 2024. More details of the public demonstration experiments are as follows:

(1) *Experimental sites:*
  (a) Zhuzi Township Farmers’ Association in Chiayi County, Taiwan;
  (b) Fenyuan Citou Community in Changhua County, Taiwan;
  (c) Zhuzi Township Library in Chiayi County, Taiwan;
  (d) Lelin Township Community Service Center in Yunlin County, Taiwan.
(2) *Participants*: Seniors aged 50 (inclusive) to 80 (inclusive) with basic cognitive and visual abilities.
(3) *Number of Participants*: A total of 52 senior participants.

### 5.1. Public Demonstrations of the System

The “Natural Rhythm through Eyes” system was primarily demonstrated at senior learning centers and other public venues. At these centers, the system was integrated into regular courses, allowing for seniors to participate during breaks. The setup included a computing device, display screens, and cameras, with venue layout featuring two poster displays and basic seating arrangements.

**Figure 23:**
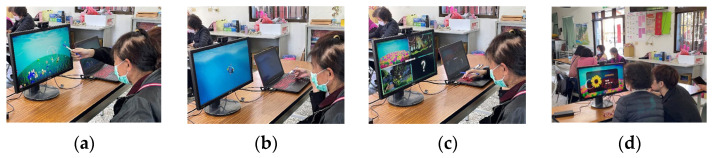
The public demonstration of “Natural Rhythm through Eyes”: (**a**) Operation explanation. (**b**) Gaze calibration. (**c**) Theme experience. (**d**) Experience results.

During the demonstrations, seniors received operational explanations, underwent gaze calibration, explored different themes, and observed the results. They interacted with the system using only their eyes, with researchers providing assistance as needed throughout the process. An overview of the “Natural Rhythm through Eyes” public demonstration process is illustrated in Figure 23.

### 5.2. Analysis of Expert Interview Results

In this study, systematic expert interviews using a semi-structured approach were conducted to interview three experts invited from relevant fields based on the research theme. One expert was in the fields of digital art and interactive multimedia, one in the field of game design, and one in the fields of elderly care and well-being. The analysis of expert interviews was conducted using the identification numbers shown in Table 5 as pseudonyms for each expert.

#### 5.2.1. Comments Collected from Expert Interviews

The content of expert interviews in this study closely adheres to the structured interview outline presented in Table 6, which encompasses three perspectives: “integration of technology into elderly activities experience”, “interactive art and active aging for the elderly”, and “application of gaze estimation in interactive art experience”. Through these perspectives, experts provided insights and recommendations regarding the overall design and experiential aspects of the proposed system “Natural Rhythm through Eyes”.

The perspective focusing on “technology integration in elderly activities experience” elicited suggestions and opinions on incorporating technological elements into the daily activities of the elderly. The perspective “interactive art and active aging for the elderly” aimed to gather perceptions of the elderly engaging in interactive art activities. Lastly, the perspective “application of gaze estimation in interactive art experience” involved discussions on integrating gaze estimation as an interactive interface in interactive art experiences, soliciting expert insights and recommendations in this area.

Based on the interview outline, experts were interviewed, and the entire process was recorded. Also, all interview content from the experts was integrated, and key recommendations for each interview question were identified and listed in Table 13 below.

#### 5.2.2. Conclusions from Expert Interviews

From Table 13, the following summary of the interviewed experts’ comments on the proposed system “Natural Rhythm through Eyes” can be drawn:

(1) The interactive interface of the system introduces a new digital experience for older adults, not only reducing physical burdens but also providing attention training.
(2) Experiencing the system can contribute to the physical and mental development of older adults, including benefits like attention training and delaying cognitive decline.
(3) The proposed system uses gaze estimation for interaction interfacing, ensuring that the system is easy to use, with real-time responses to the elderly’s actions.
(4) The proposed system can incorporate interactive elements that better align with older adults’ past experiences and feature a simple and bright interface design.
(5) The system can introduce timely interactive processes akin to overcoming challenges, providing enjoyment from immediate feedback to enhance older adults’ sense of participation and satisfaction.

### 5.3. Analysis of Questionnaire Survey Results

After the participants completed the process of system experiencing, they were asked to fill out a questionnaire. Depending on the individual circumstances of the older adults, assistance was provided in filling out the questionnaire. A total of 52 valid questionnaires were collected in this study. In addition to the “basic information” about the users, the questionnaire survey content includes three more parts about the assessment of the proposed system “Natural Rhythm through Eyes” from the users’ viewpoint: “user friendliness”, “user satisfaction”, and “user experience”.

#### 5.3.1. Sample Structure Analysis

The first part of the questionnaire collected basic information about the participants as well as the statistics of three main aspects, gender, age, and prior experience with similar technological products, as shown in Table 14. Accordingly, females constituted 44% of the participants, males 8%, with an average age of 60 years. Regarding prior experience with similar technological products, 63% reported almost no prior usage, 29% reported occasional usage, and 8% reported frequent usage. Hence, it is evident that the majority of elderly participants had had little exposure to similar technological products.

#### 5.3.2. Analysis of Reliability and Validity of Questionnaire Survey Results

Before assessing the effectiveness of the proposed system, “Natural Rhythm through Eyes”, the *reliability* and *validity* of the collected questionnaire data were verified by use of the software packages IBM SPSS and AMOS.

Firstly, the questionnaire data of the three scales of *user friendliness*, *user satisfaction*, and *user experience* were assigned the scores 1 through 5 according to the Likert five-point scale, with each item in the questionnaire referring to the numbering in Table 7, Table 8 and Table 9. The results are shown in Table 15, Table 16 and Table 17, respectively, in which the minima (Min.), maxima (Max.), means, and standard deviations (S. D.) of the data are also listed. Then, the process of verifying the reliability and validity of the questionnaire data was conducted sequentially in five steps, as described in the following:

(1) *Step 1* *: Verification of the adequacy of the questionnaire dataset*

The *adequacy* of the collected questionnaire data had to be verified first. For this purpose, the Kaiser–Meyer–Olkin (KMO) test and Bartlett’s test of sphericity [105] were adopted in this study. A KMO value exceeding the threshold of 0.50 is generally considered to pass the KMO test, while a significance value of Bartlett’s test below the threshold of 0.05 is typically accepted to pass the test. When both tests are passed, the dataset is thought *adequately related*, indicating its *adequacy* for further structural analysis.

Specifically, using the data from Table 15, Table 16 and Table 17 as inputs for the SPSS, the computed KMO values and Bartlett’s significant values were determined, presented in Table 18. Accordingly, it is evident that all the three KMO measure values exceed 0.50, and the three computed significant values for Bartlett’s test are below 0.05, meaning that both tests were passed and that the data from Table 15, Table 16 and Table 17 are *suitable for further structural analysis*, as determined in the subsequent steps of data verification.

(2) *Step 2* *: Finding the latent dimensions of the questions from the collected data*

In the structural analysis of questionnaire data, the aim is to categorize the questions of each scale into meaningful subsets, with each subset being associated with a *latent dimension*. For this, the *exploratory factor analysis* (EFA) utilizing *principal component analysis*, along with *the varimax method with Kaiser normalization*, was applied by use of the SPSS. The outcomes of these procedures, with Table 15, Table 16 and Table 17 as inputs, are presented in Table 19, Table 20 and Table 21 for the three scales of *user friendliness, user satisfaction,* and *user experience*, respectively.

Specifically, for the first scale of user friendliness, the variables R1 through R8, representing the ten questions asked about this scale, fall into three groups, RC1 = (R1, R3, R6, R2), RC2 = (R7, R8, R5, R4), and RC3 = (R7, R8), aligned with three latent dimensions termed in this study to be “intention to act”, “usefulness”, and “usability”, respectively. Likewise, for the second scale of user satisfaction, the results are the two groups SC1 = (S7, S8, S2, S10, S1) and SC2 = (S6, S4, S6, S3, S5) of two latent dimensions termed “pleasure and relaxation” and “cognitive stimulation”, respectively. Finally, for the third scale of user experience, the results are the two groups TC1 = (T2, T1, T8, T8, T3, T4) and TC2 = (T5, T6, T4, T7) of the latent dimensions termed “experience satisfaction” and “enrichment in life”, respectively. These results are summarized integrally in Table 22.

(3) *Step 3* *: Verifying the reliability of the collected questionnaire data*

Reliability refers to the consistency of a dataset across multiple repetitions [106]. In this study, the analysis of the reliability of the collected questionnaire data is based on the Cronbach’s α coefficient [107] that is yielded by the EFA mentioned previously. It is recognized that as the Cronbach’s α coefficient approaches the extreme value of 1.0, the reliability of the dataset (considered as variables) increases. A Cronbach’s α coefficient exceeding the threshold of 0.35 indicates that the data in question are reliable, and a value surpassing 0.70 signifies high reliability of the dataset [108].

Utilizing the data presented in Table 15, Table 16 and Table 17, the Cronbach’s α coefficients for the three scales and the six latent dimensions are detailed in Table 23. It is evident from the table that all Cronbach’s α coefficients surpass 0.70. Consequently, the collected questionnaire datasets for the three scales and the individual latent dimensions are considered *reliable for further analysis*.

(4) *Step 4* *: Verification of applicability of the structural model established with the dimensions*

**Figure 24:**
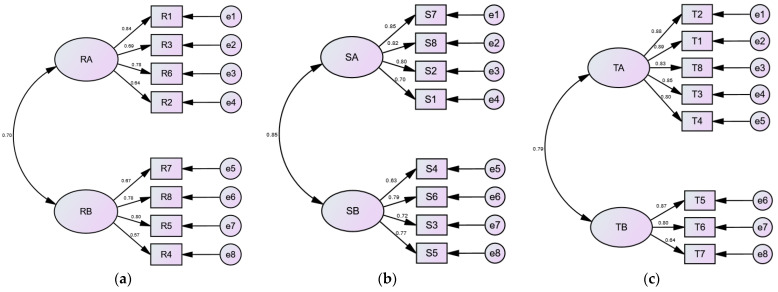
Results of confirmatory factor analysis (CFA) using the AMOS package: (**a**–**c**) the structural models of the scales of “user friendliness”, “user satisfaction”, and “user experience” generated through CFA, respectively.

Before establishing the validity of the questionnaire data, it is essential to verify the appropriateness of the structural model configured based on the latent dimensions. To accomplish this, the *confirmatory factor analysis* (CFA) process, utilizing the AMOS package, was applied to the gathered questionnaire data. The outcome of this analysis resulted in three structure model graphs, as depicted in Figure 24. Additionally, the CFA generated a comprehensive list of *structure-model fit indices* for each of the three scales, “user friendliness”, “user satisfaction”, and “user experience”, as presented in Table 24. It can be seen that the three index values of χ^2^/df, cfi, and RMSEA obtained for each scale are, respectively, “between 1 to 5”, “larger than 0.9”, and “approximately between 0.05 and 0.08”, indicating that the structural model, established according to the latent dimensions of the scale, *fits fairly* to the collected questionnaire data according to Hu and Bentler [109].

(5) *Step 5* *: Verification of the validity of the collected questionnaire data*

Having established the fact that the model structures of the three scales fairly fit the questionnaire data, the next step involved the analysis of the data’s *validity*. In Figure 24 of the structure model of the questionnaire data, all the *factor loading values* (standardized regression weights) related to the three scales (along the paths from RA and RB to questions R1 through R8, SA and SB to questions S1 through S7, and TA and TB to T1 through T8) exceed the threshold 0.5. This observation indicates that the *construct validity* of the model is fairly good. Furthermore, this validation is reinforced by the construct validity values of all latent dimensions computed by the EFA process mentioned earlier and detailed in Table 25, where each value surpasses the threshold of 0.6 for passing the construct validity verification.

Through the above five steps of statistical processes, the verification of the reliability and validity of the questionnaire data was completed. We then proceeded to analyze the questionnaire data of each latent dimension, as shown subsequently.

#### 5.3.3. Analysis of Questionnaire Data about the Scale of User Friendliness

The scale of user friendliness in this study was designed to investigate older adults’ perspectives on the usability and acceptance of gaze estimation technology in their technological applications after interacting with the system. The assessment scale was crafted following the technology acceptance model (TAM), encompassing two latent dimensions: “intention to act” and “usefulness”. A concise overview of the results is described in the following:

(A) *Data analysis for the latent dimension “intention to act”*

The main focus was on exploring post-experience behavioral performance, willingness to use, and acceptance level. The analysis of the latent dimension “intention to act” is shown in Table 26. Based on the table, the following points are summarized and analyzed:

(1) The average values of the dimension “intention to act” are all greater than 3.5, with agreement rates exceeding 60%, indicating that a majority of the older adults expressed a certain level of recognition and acceptance towards interacting with and using the estimated gaze interface.
(2) Question R1 and R6 have average values above 4, indicating that the older adults were satisfied with the accuracy of the gaze estimation system. Through this interactive interface, older adults were more willing to engage with technology. However, a small percentage of older adults disagreed, believing that the interactive interface was not easy to operate smoothly.
(3) The average vagrealues for items R3 and R2 are both below 4, but the agreement rates exceed 60%. This indicates that some older adults showed no significant interest in the system and did not intend to incorporate its use into their daily leisure activities. A few individuals even expressed disagreement with these statements.

(B) *Data analysis for the latent dimension “usefulness”*

This latent dimension mainly explored how older adults perceived the functionality of the system after experiencing it. The analysis of the latent dimension “usefulness” is shown in Table 27. Based on the table, the following points can be summarized:

(1) The average values of this dimension are all greater than 4, with agreement rates exceeding 70%. This indicates that a majority of the older adults find gaze estimation helpful for their use of technology.
(2) Question R4 has a standard deviation close to 1, and both R4 and R5 show some disagreement options, suggesting divergent views among the older adults regarding the convenience of gaze estimation and its effectiveness in reducing physical burden when operating technology. However, with agreement rates exceeding 70% and average scores above 4, it shows that most of the older adults perceived the system interface as beneficial for their use of technology.
(3) Questions R7 and R8 have zero percentages for disagreement and strong disagreement, with agreement rates exceeding 80%, indicating that the older adults found the gaze estimation operations simple and felt confident during the experiencing process.

#### 5.3.4. Analysis of Questionnaire Data about the Scale of User Satisfaction

The scale of “user satisfaction” in this study aimed to understand whether the older adults exhibited positive emotions during the system experiencing process. This assessment scale was designed with reference to the aesthetic emotion scale (AESTHEMOS). The final classification included two latent dimensions: “pleasure and relaxation” and “cognitive stimulation”. Below, a brief overview of these two latent dimensions are presented in sequence:

(A) *Data analysis for the latent dimension “pleasure and relaxation”*

The dimension of “pleasure and relaxation” primarily explored whether the older adults enjoyed the visual presentation of interactive art after experiencing the system, whether they were attracted to it, and whether they exhibited pleasurable emotional responses during the process, as well as whether there was a role in emotional regulation. The analysis results of this latent dimension are shown in Table 28, based on which the following points are summarized:

(1) The average values of this latent dimension are all greater than 4, with agreement rates exceeding 80%, indicating that the majority of the older adults exhibited positive emotions during the system experience process.
(2) Question S8 and S1 have standard deviations below 0.7, and agreement rates exceeding 90%, indicating that the older adults were satisfied with the system’s visual design and interactive content.
(3) Question S7 has a standard deviation above 0.8 and includes disagreement options, suggesting that some older adults experienced a sense of relaxation during the experience. However, overall, the relaxed pace contributed to calming their emotions.
(4) Question S2 and S1 have zero percentages for disagreement and strong disagreement, indicating that the older adults perceived the visual presentation as aesthetically pleasing and mood-enhancing.

(B) *Data analysis for the latent dimension “cognitive stimulation”*

The latent dimension “cognitive stimulation” primarily explored the emotional experiences of the older adults at a spiritual level following their system experience process, prompting them to seek experiential meaning or be emotionally moved to action. The analysis results of this latent dimension are presented in Table 29, based on which the following points are summarized:

(1) Questions S4 and S3 have standard deviations above 0.8, with disagreement options present. Among these, S4 have average scores below 4, indicating that for some older adults, the interactive content may have seemed somewhat bland, failing to create an immersive or memorable experience.
(2) Question S5 shows a higher percentage of agreement, suggesting that the exploratory interactive content piqued curiosity among the older adults.
(3) Question S7 has no disagreement or strong disagreement, indicating that overall, the older adults exhibited emotionally driven responses to action following their system experience.

#### 5.3.5. Analysis of Questionnaire Data about the Scale of User Experience

The scale of “*user experience*” aimed to understand if the older adults perceived an enhancement in their quality of life following their system experience. The assessment scale was designed based on the experience strategy module (SEMs), categorizing outcomes into two latent dimensions: “experience satisfaction” and “enrichment in life”. Below are summarized descriptions of these two latent dimensions in sequence:

(A) *Data analysis for the latent dimension “experience satisfaction”*

The latent dimension *experience satisfaction* was primarily used to explore whether the older adults experienced positive benefits after using the system, including physical, mental, and emotional relief, or found the system experience meaningful, enjoyable, or refreshing. It comprehensively evaluated the older adults’ satisfaction with the system experience. The analysis results of this dimension are summarized in Table 30, outlining the following points:

(1) The average scores for the “experience satisfaction” dimension are all above 4, with no responses indicating disagreement or strong disagreement. This indicates that the majority of older adults, after experiencing the system, were satisfied with the benefits it brought to them.
(2) Question T8 shows a proportion of agreement exceeding 90%, with a standard deviation below 0.7. This suggests that most of the older adults perceived the system as beneficial to enhancing their social participation.
(3) Question T3 has a relatively high proportion of responses in the neutral category, indicating that for some older adults, there was less noticeable relief from stress after experiencing the system.

(B) *Data analysis for the latent dimension “enrichment in life”*

The latent dimension of “enrichment in life” primarily explored the extent to which the older adults perceived the system’s impact on their daily lives and whether it enhanced their positive emotions towards life. This included connecting life experiences, expanding daily life knowledge, and stimulating social interactions, enriching various aspects of the older adults’ daily lives. The analysis results for this latent dimension, as shown in Table 31, are summarized as follows:

(1) The average scores for this latent dimension are all above 4.0, indicating that the majority of the older adults believed that the activities experienced had a positive impact on their lives.
(2) Question T7 has a higher proportion of neutral responses, indicating that for some the older adults, the system did not stimulate an active mindset towards social interaction.

#### 5.3.6. A Summary of Questionnaire Survey Data Analysis

This study synthesized the expert interview results from the table above, concluding the following about the proposed interactive system “ Natural Rhythm through Eyes”:

(1) The integration of gaze estimation with interactive art activities has a positive impact on the quality of life factors for older adults, including their physical and mental development and social interactions.
(2) Integration of gaze estimation with interactive art positively impacts older adults’ quality of life, enhancing physical and mental development, and social interactions.
(3) Abstract visual designs may not effectively stimulate active responses in older adults due to their diverse life backgrounds.
(4) Diverse themes and interactive elements provide more opportunities for older adults to engage with systems, sparking curiosity and enhancing enjoyment.
(5) Older adults highly accept visual designs themed around nature, creating a relaxing emotional state.
(6) Lack of challenging interactive elements in system design leads to varying perceptions among older adults regarding immersion and engagement.
(7) Older adults find eye-interaction interfaces convenient and beneficial for physically aging populations.
(8) Stringent pre-calibration procedures increase learning burden for older adults, impacting system usability and user acceptance.

## 6. Conclusions and Suggestions

### 6.1. Discussion and Contributions

Based on expert interviews, suggestions for improving the proposed system “Natural Rhythm through Eyes” were identified. Overall, the older adult users provided predominantly positive feedback on their experience with the system. They viewed the gaze estimation interface as beneficial for enhancing motivation to learn and engage with technology. Additionally, the interactive art system elicited positive emotions among the older adults, indicating its effectiveness in promoting active aging.

Through analyzing expert interviews and survey results, several key findings were identified:

(1) Older adults perceive the proposed system “Natural Rhythm through Eyes” positively for its interaction methods and social inclusivity, indirectly supporting attention training.
(2) Usability of the proposed system is noted to be relatively low, highlighting areas for improvement in gaze estimation calibration and operational feedback.
(3) The natural ecological visual design of the proposed system relaxes older adults emotionally, while its interactive format stimulates enjoyable engagement.
(4) Older adults reported positive emotional experiences with the proposed system, enhancing their quality of life.
(5) The proposed system lacks challenging interactive elements, which could be enhanced to increase older adults’ participation and satisfaction.
(6) Integration of interactive elements in the proposed system aligns with older adults’ past experiences, strengthening life connections.

These findings collectively underscore the potential of the proposed system “Natural Rhythm through Eyes” in positively impacting the lives of older adults, while also highlighting areas for refinement to enhance usability and engagement.

### 6.2. Conclusions

How integrating gaze estimation technology with interactive art systems promotes active aging among older adults was explored in this study. Through a literature review and case analysis, the physiological and psychological needs of older adults were examined to develop key activities for active aging.

Gaze estimation technology was utilized as the system’s interaction interface to strengthen the connection between older adults and technology through simple eye movements. Interactive art employed various sensory experiences and themes to stimulate rich emotional expressions, fostering positive emotions and potentially delaying cognitive decline. Integrating these aspects into a soothing experiential rhythm allowed for older adults to enjoy meaningful activities, benefiting from active engagement and promoting quality of life.

The proposed interactive system “Natural Rhythm through Eyes” was showcased in a public exhibition, inviting older adults to participate and provide feedback through a questionnaire survey. Expert interviews were also conducted. The evaluation aimed to assess usability, pleasure, and interactive experience, demonstrating that integrating gaze estimation and interactive art can promote active aging. Based on the evaluation, this study concludes that integrating gaze estimation with interactive art effectively supports active aging among older adults.

More specifically, the following conclusions can be drawn in this study from using the proposed system “Natural Rhythm through Eyes”:

(1) *Enhanced Usability of Gaze Estimation for Elderly Users’ Convenience in life*

Gaze estimation technology was employed in this study to enable system control through eye movements. Surveys and expert interviews revealed that older adults found the interface to be convenient, user-friendly, and inclusive.

(2) *Increased Enjoyment and Engagement Through Nature-Themed Interactive Art*

Questionnaire surveys indicate that older adults had positive experiences with system designs based on natural themes. Interaction with nature offers psychological and health benefits, such as stress relief, which positively affected their well-being. Additionally, exploratory interaction formats elicit enjoyable emotions through engaging and attractive content.

(3) *Positive Influence on Active Aging Through the Integration of Gaze Estimation and Interactive Art*

This study combined gaze estimation technology with interactive art to create the system “Natural Rhythm through Eyes”. The results showed that the majority of older adults rated the system highly after using it. The system performed well in interaction experience and pleasure indices, providing positive emotional experiences and enhancing overall quality of life.

### 6.3. Suggestions for Future Studies

The main aim of this study was to explore active aging among older adults and develop interactive systems tailored for them. Based on feedback from expert interviews and surveys, this study suggests improvements and future directions considering current constraints. Key recommendations and future research directions include the following:

(1) Simplifying the calibration process of the gaze estimation interactive interface and enhancing user guidance through visual feedback using animations and sound.
(2) Enhancing the system’s relevance to older adults’ lives to increase satisfaction, such as incorporating nostalgic music and bright, simple interfaces.
(3) Introducing challenging interactive content within feasible limits to promote active engagement and immersion.
(4) Enriching the system interface with animations, images, and interactive feedback to diversify themes and attract attention, thereby improving usability.
(5) Expanding the application of the developed interactive system to various settings for a broader range of older adults, modularizing the system based on different experiential contexts.
(6) Introducing multiplayer interaction modes to enhance social engagement among older adults, allowing for interaction with friends and family to foster a sense of belonging and satisfaction.
(7) Regarding the evaluation of the effectiveness of the proposed system, it may be beneficial to include a sample of older adults as a control group. This would allow for analysis and comparison of the differences between the experimental group and the control group to reach a more precise conclusion.

These recommendations aim to enhance the usability and effectiveness of interactive systems designed for promoting active aging among older adults.

## Figures and Tables

**Table 1:** Summary of studies related to healthy aging.

Theory	Year	Scholar/Organization	Main Content
**Healthy Aging**	1990	WHO	Maintaining healthy physical and mental functions, including complete normalcy in physiological, psychological, and social aspects.
**Successful Aging**	1987 and 1997	Rowe and Kahn	Avoiding the occurrence of diseases and disabilities, maintaining good cognitive abilities and physical functions, and actively participating in life.
	1990	Baltes and Baltes	The process of good psychological adaptation in older adults, covering three elements: selection, optimization, and compensation, abbreviated as the SOC model.
	1999	Torres	Perspectives considering cultural factors, including five value tendencies: human nature, human–nature relationships, interpersonal relationships, time, and activities.
**Robust Aging**	1995	Garfein and Herzog	The robustness of body functions, mental health, cognitive functions, and participation in productive activities.
**Gerotranscendence**	1997	Tornstam	Promoting a positive attitude towards aging, advocating for an optimistic, natural attitude to re-evaluating everything in life.
**Active Aging**	2002	WHO	Enjoying life in a healthy, participatory, and safe manner during the aging process, thereby enhancing the quality of life for older adults.
**2020–2030 Decade of Healthy Aging Action**	2020	WHO	Aiming to achieve the noble goal of “Leaving No One Behind”, based on four action areas: eliminating age discrimination, building age-friendly environments, comprehensive health care, and long-term care.

**Table 2:** Compilation of orange technology applications for the elderly.

Case	Orange Technology Type	Orange Computing Type	Presentation Form	Explanation
PARO (2022) [51]	Happiness technology	Affective computing	Robot	Generating autonomous responses to stimuli, conducting learning, and interacting with users.
Zenbo (2016) [52]	Happiness technology	Affective computing	Robot	Providing education, personal assistance, and entertainment through voice commands.
PECOLA (2022) [53]	Health technology, warm technology	Biological signal processing and health information	Robot	Applying facial recognition to assist the elderly with diet analysis, sleep monitoring, and issuing fall alerts.
RoBoHoN (2022) [54]	Happiness technology, warm technology	Advanced companion or assistive technology	Robot	Supporting facial recognition with projectors and cameras for emergency alerts and displaying emotional expressions.
ELLI.Q (2022) [55]	Health technology, happiness technology	Advanced companion or assistive technology	Robot	Monitoring health, scheduling activities, and serving as an assistive companion for the elderly.
A Socially Assistive Robot for Elderly Exercise (2019) [56]	Health technology	Biological signal processing and health information	Robot	Implementing a deep learning-based exercise system for senior sports training.
Biostamp (2012) [57]	Health technology	Biological signal processing and health information	Wearable device	Featuring a stretchable electronic patch design for remotely monitoring users’ vital signs.
CARE System (2017) [58]	Happiness technology	Affective computing	Interactive device	Retrieving useful information from family photos to recommend activities for elderly users.

**Table 3:** Compilation of interactive art applications for the elderly.

Case	Presentation Form	Interactive Form	Sensory Experience	Description
Tovertafel (2015) [72]	Interactive table	Contact interaction	Vision, hearing, touch	Interactive projection devices tailored for elderly dementia patients, featuring multiplayer games with unlimited user participation.
Living Aquarium (2022) [73]	Interactive wall	Contact interaction	Touch, vision, hearing	Dual-projector virtual aquarium simulating underwater coral life, interacted with via hand gestures by the elderly.
Digital Art Installation with Cognitive and Motivation Scores (2020) [11]	Interactive space	Distance interaction	Vision, hearing	Home-based digital art installation with seven scenes, employing depth cameras and microphones to capture elderly movements and sounds, presenting imaginative visual feedback.
VENSTER (2017) [12]	Interactive table	Contact interaction	Vision, hearing, touch	Window-shaped art installation with standby, trigger, and interactive modes, allowing for touch-based interaction.
Interactive Sensory Projection Systems (2022) [74]	Interactive table, wall, and Floor	Distance interaction and contact interaction	Vision, hearing, touch	Three distinct interactive systems offering varied interaction options, primarily through physical activity.
FishCatcher (2013) [75]	Monitor	Distance interaction	Vision, hearing	Color-catching fish game using hand gestures for interaction, promoting multiplayer cooperation.
Rassle (2018) [76]	Robot monitor	Contact interaction	Vision, hearing, touch	Teddy bear-shaped robot integrating tactile sensing for exercise and offering auditory and visual experiences to engage elderly users.

**Table 4:** Compilation of gaze estimation applications for the elderly.

Case	Sensing Device	Technical Application	Application Content	Description
Eye Tracking-Based Assistance System (2019) [19]	Camera	IoT, image processing, micro-control device	Smart furniture	Integrating gaze estimation, speech recognition, and IoT for interacting with smart furniture in the living space.
Look to Speak (2020) [88]	Mobile device	Deep learning on Android	Instant messaging	Developing an Android app based on gaze estimation to aid users in interacting with others with no barriers.
Eye-Tracking 3D MUI (2015) [89]	VR device, camera	Image processing, VR	Multimedia functionality and interactive gaming	Creating a multimedia user interface in a 3D environment, utilizing VR with cameras to track eye movements.
HCI Control System of Wheelchairs (2021) [90]	Infrared light filter camera	Deep learning, image processing, micro-control device	Smart wheelchair	Allowing for users to control the direction of the wheelchair through the position of the head and the direction of gaze.
Beam (2022) [91]	Mobile device	Deep learning on iOS	Interactive gaming	Utilizing Apple phones to predict gaze positions, allowing for users to utilize phone features and experience gaming fun.
EyeMine (2018) [92]	Eye tracker	Image processing	Interactive gaming	Developing gaze estimation for the Minecraft game, supporting various eye-tracking devices.
StarGaze (2018) [93]	Eye tracker	Image processing	Multimedia and interactive gaming	Designing a gaze estimation interface for in-bed computer operation for patients due to trauma or severe conditions.
Robotic System for Painting with Eyes (2021) [94]	Eye tracker	Signal processing, image processing, robot arm	Artistic creation	Using gaze estimation with robotic arms to achieve artistic painting and creation goals by controlling the robotic arm through gaze trajectories.
Hybrid Brain–Computer Interface (2021) [95]	EEG, headset retinal camera	Brainwave analysis, image processing, force feedback robotic arm	Assistive technology	Using a hybrid brain–computer interface, eye electrical signals, gaze estimation, and force feedback to operate a robotic arm for object manipulation.

**Table 5:** Experts invited for interviews in this study.

Expert No.	Position	Title	Expertise
Q1	National University	Professor	Cross-domain integrated design, value-added design for picture books, digital imaging, photography
Q2	National University	Professor	Image processing, game design, human–computer interaction development
Q3	Elderly Learning Center	Lecturer	Elderly care and well-being, activity planning and management, digital learning education design

**Table 6:** Outline for expert interviews.

Aspect	Interview Questions
Technology integration in elderly activities experience	Is integrating interactive systems suitable for elderly activity experiences?
	How can interest be generated among the elderly for the interactive system?
Interactive art and active aging for the elderly	Can interactive art experiences positively contribute to the physical and mental development of the elderly?
	What are your thoughts and suggestions on designing interactive art experience themes and scenarios for the elderly?
Application of gaze estimation in interactive art experience	What are your views on using gaze estimation as an interactive interface for elderly interactive art experiences?
	What details should be considered when developing systems using gaze estimation technology?

**Table 7:** Questionnaire design for the scale of user friendliness.

Question No.	Content
R1	I am more willing to try technology through eye interaction.
R2	I believe that eye interaction can capture my interest.
R3	In my leisure time, I think I will use this system frequently.
R4	I feel that interacting through eyes helps alleviate physical burden.
R5	I think using eye interaction makes the system more convenient.
R6	I believe that system interactions via eyes can meet functional requirements.
R7	I find eye interaction very easy.
R8	I am very confident in using the system.

**Table 8:** Questionnaire design for the scale of user satisfaction.

Question No.	Content
S1	I really like the natural scenery presented in the visuals.
S2	I find the natural scenery presented in the visuals very beautiful.
S3	The experience process leaves a deep impression on me.
S4	I can easily immerse myself in the experience process.
S5	The interactive feedback makes me curious.
S6	After the experience, I feel more energetic.
S7	I feel very relaxed during the experience process.
S8	I find the experience content interesting.

**Table 9:** Interactive experience assessment questionnaire design.

Question No.	Content
T1	This experience activity is very interesting to me.
T2	I feel that this experience activity helps me relax.
T3	I feel that this experience activity helps me relieve stress.
T4	I feel that this is a meaningful experience activity.
T5	This experience activity enriches my daily life.
T6	I have a better understanding of the use of technology after system experiencing.
T7	After the experience ends, I feel more active in interacting with others.
T8	I think it helps to increase willingness to participate in activities after system experiencing.

**Table 10:** Conceptual illustrations of dynamic visual design in the interactive art system.

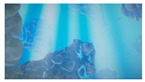	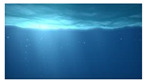	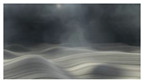	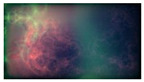
Deep sea	Shallow sea	Desert	Starry sky
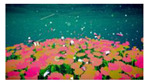	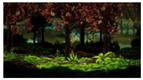	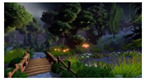	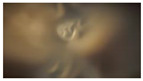
Garden	Forest	Rainforest	Cloud layer

**Table 11:** The theme interaction contents of the proposed interactive art system.

Theme	Interactive Species	Species Type	Symbolic Meaning	Illustration
**Star Garden**	Flowers	Sunflower	Hope, courage, vitality	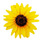
		Platycodon	Nobility, loyalty, beauty	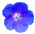
		Carnation	Purity, friendship, elegance	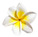
		Rose	Passion, beauty, renewal	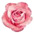
**Animal Forest**	Animals	Brown bear	Strength, protection, calm	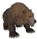
		Deer	Gentleness, sensitivity, beauty	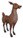
		Stag	Wisdom, leadership, grace	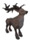
		Caribou	Resilience, courage, sturdiness	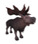
**Forest Trees**	Trees	Cedar	Longevity, resilience, protection	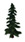
		Birch	Vigor, youth, vitality	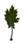
		Pine	Tranquility, steadfastness, evergreen	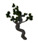
		Cypress	Resilience, stability, nobility	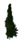

**Table 12:** Illustrations of the process of the proposed system “Natural Rhythm through Eyes”.

Stage	Process	Illustration	Eye Move	Explanation
**Stage 1**	**Head Adjustment**	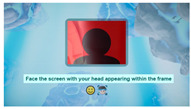	No	In the effective range, when there are no users detected, the mask in the camera view will display in red.
		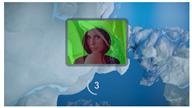		When a user approaches the system and places his/her face within the mask, the mask will display in green. If it remains green, a five-second countdown will begin, after which the formal experience will commence.
**Stage 2**	**Gaze Calibration**	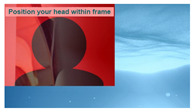	No	When the system cannot detect the user’s face within the mask position, the mask in the camera view will display in red and appear in the top-left corner. At this point, the system will pause operation until the user adjusts their head to a valid position.
		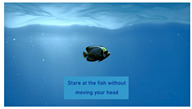		The user is required to follow system prompts and keep their gaze fixed on the fish without moving their heads. After a 3 s countdown, the system initiates calibration.
		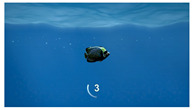		The fish moves rhythmically across the screen, pausing for several seconds in each corner over time. As the user tracks the fish with his/her gaze, the system captures the user’s eye movement data during the fish’s pauses.
		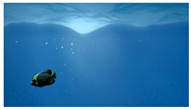	Smooth Pursuit	The fish moves rhythmically across the screen, pausing for several seconds in each corner over time. When the user tracks the fish with his/er gaze, the system captures the user’s eye movement data during these pauses.
**Stage 3**	**Gaze Testing**	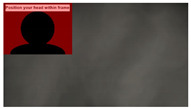	No	When the system cannot detect the user’s face within the mask, the mask will display in red and appear in the top-left corner. At this point, the system pauses operation until the user adjusts his/her head to a valid position.
		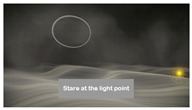		Operational prompts are displayed, and the user is required to follow these instructions. A white heatmap indicates the estimated gaze position of the user during the gaze estimation process.
		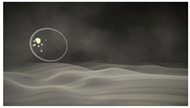	Reactive Saccades	The user moves his/her gaze to randomly appearing light spots on the screen. When his/her gaze touches a spot, it will disappear, and the next spot will appear immediately.
		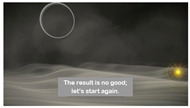	No	If not all spots are successfully triggered within the limited time in this stage, the system will return to the second stage for gaze estimation recalibration.
**Stage 4**	**Topic Selection**	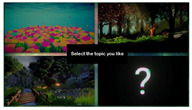	No	The screen is evenly divided into previews of three themes. The top-left is the Star Garden theme, the top-right is the Animal Forest theme, the bottom-left is the Forest Trees theme, and the bottom-right is random.
		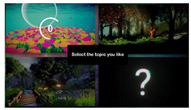	Fixations	The user gazes at the area of the theme image he/she wishes to select. After one-second fixation on the chosen theme, he/she will enter theme experiencing. Selecting the question mark will enter one of the main themes.
**Stage 5**	**Theme Experience—Star Garden**	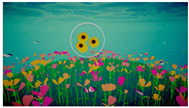	Fixations	While gazing at the screen, a random flower will bloom after one second of fixation, spreading outwards from the fixation point.
		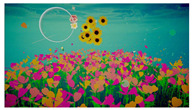	Scanning Saccades	When the user starts moving his/her gaze, the flowers of that type will scatter.
		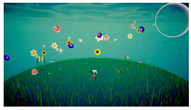		If the user drags his/her gaze extensively, all four types of flowers will elegantly bloom along the trajectory.
		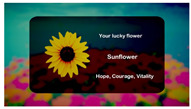	No	The system analyzes the total fixation duration for each type of flower to determine the most focused flower type and its symbolic meaning for the theme. If the user has experienced all themes, go to Stage 6; else, return to Stage 3.
	**Theme Experience—Animal Forest**	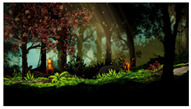	No	In the scene, various animals will randomly appear, each exhibiting their own behaviors. The camera perspective will move back and forth from right to left.
		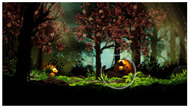	Fixations	When the user gazes at an animal, the animal will perceive this and react accordingly.
		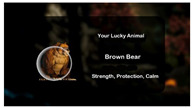	No	Comparing the durations of gaze fixation on various animal types, the theme will attribute significance to the animal type receiving the most attention. If all themes have been experienced, go to Stage 6; else, return to stage 3.
	**Theme Experience—Forest Trees**	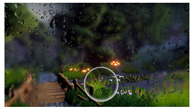	Scanning Saccades	The user’s gaze focus area can erase the scenery outside the mist observation window. The camera perspective will move back and forth from near to far.
		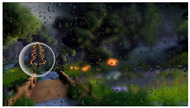	Fixations	When the user gazes at specific types of trees in the scene, those trees will be marked and sway.
		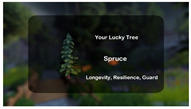	No	Comparing the durations of gaze fixation on various types of trees, the theme will attribute significance to the tree type receiving the most attention. If all themes have been experienced, the system proceeds to Stage 6; else, it returns to Stage 3.
**Stage 6**	**Experience Results**	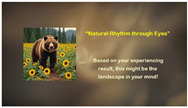	No	Based on the user’s identified focus species from the experiences in the three themes, the names of these three species are used as keywords to generate a natural landscape image containing them on the screen.

**Table 13:** Results of expert interviews in this study.

Interview Aspect	Interview Question	Expert’s Comment
Integration of technology into elderly activities	Is integrating interactive systems suitable for elderly activity experiences?	The gaze estimation system benefits seniors with limited mobility by minimizing the need for physical movements. System design should prioritize creating an easy-to-learn, user-friendly environment to reduce psychological barriers for users new to technology. Optimizing interface design and system operations for the elderly is crucial to enhancing their overall experience and enjoyment, not just to facilitating learning.
	How can interest be generated among the elderly for interactive systems?	Including music styles and classic melodies from the elderly’s youth enhances interest and emotional connections. Designing simple, bright interfaces that cater to visual needs and incorporating relaxed, playful interactive elements increase attractiveness. Implementing interactive processes with challenges enhances the overall experience, allowing for the elderly to enjoy a sense of achievement in gaming.
Interactive art and active aging for the elderly	Can interactive art experiences positively contribute to the physical and mental development of the elderly?	It can significantly help older adults to train their attention and focus, contributing to the delay in cognitive decline. It can provide psychological fulfillment and emotional connections, not only stimulating mentally but also enhancing social interaction. Gamified interactive designs can transform learning into a pleasant and engaging experience, effectively delaying cognitive decline.
	What are your thoughts and suggestions on designing interactive art experience themes and scenarios for the elderly?	When designing themes, it is suggested to use bold and contrasting colors, as well as clear images, to make it easier for the elderly to identify and understand. Themes can revolve around natural elements familiar and beloved by older adults, such as target species. Background visual complexity should be avoided, and the visual contrast of target species should not be too weak. Visual context design can consider incorporating elements that resonate with older adults’ past life experiences, fostering a sense of connection.
Application of gaze estimation in interactive art experience	What are your views on using gaze estimation as an interactive interface for elderly interactive art experiences?	The application of gaze estimation technology as an interactive interface provides a novel digital interaction method, representing a promising endeavor. Interacting through eye movements without requiring any manual actions is particularly beneficial for older adults with limited physical abilities. In the design of interactive art, expanding the range of interface interactions, like controlling perspectives, allows for users to experience a more liberated space.
	What details should be considered when developing systems using gaze estimation technology?	In terms of technical development, it is important to ensure that the accuracy and response speed of gaze tracking meet the usage habits of older adults. Object triggers in theme experiences should include zoom-in capabilities, making it easier for the elderly to observe details and enhance interactive joy. Interactive feedback in the interface can be enriched with abundant sound cues and animated responses, making it more attractive and facilitating older adults’ enjoyment of the entire experience process.

**Table 14:** Statistics of basic data of sample participants.

Basic Data	Categories	Number of Samples	Percentage
Sex	Male	8	15%
	Female	44	85%
Age	50–60	13	25%
	61–70	18	35%
	71–80	15	29%
	over 80	6	11%
Ever used similar technological products?	Frequently	33	8%
	Sometimes	15	29%
	Almost never	4	63%

**Table 15:** Statistics of the scale of “user friendliness” questionnaire data.

Item No.	Min.	Max.	Mean	S. D.	Strongly Agree	Agree	Neutral	Disagree	Strongly Disagree	Agree or Above
R1	3	5	4.12	0.732	32.7%	46.2%	21.2%	0.0%	0.0%	78.9%
R2	3	5	3.98	0.700	23.1%	51.9%	25.0%	0.0%	0.0%	75.0%
R3	3	5	4.12	0.732	32.7%	46.2%	21.2%	0.0%	0.0%	78.9%
R4	2	5	4.04	0.928	38.5%	32.7%	23.1%	5.8%	0.0%	71.2%
R5	2	5	4.21	0.720	38.5%	46.2%	13.5%	1.9%	0.0%	84.7%
R6	2	5	4.10	0.721	28.8%	53.8%	15.4%	1.9%	0.0%	82.6%
R7	3	5	4.27	0.660	38.5%	50.0%	11.5%	0.0%	0.0%	88.5%
R8	3	5	4.21	0.723	38.5%	44.2%	17.3%	0.0%	0.0%	82.7%

**Table 16:** Statistics of the scale of “user satisfaction” questionnaire data.

Item No.	Min.	Max.	Mean	S. D.	Strongly Agree	Agree	Neutral	Disagree	Strongly Disagree	Agree or Above
S1	3	5	4.37	0.658	46.2%	44.2%	9.6%	0.0%	0.0%	90.4%
S2	3	5	4.38	0.718	51.9%	34.6%	13.5%	0.0%	0.0%	86.5%
S3	2	5	4.08	0.813	34.6%	40.4%	23.1%	1.9%	0.0%	75.0%
S4	2	5	3.75	0.813	21.2%	34.6%	42.3%	1.9%	0.0%	55.8%
S5	3	5	4.31	0.701	44.2%	42.3%	13.5%	0.0%	0.0%	86.5%
S6	3	5	4.06	0.777	32.7%	40.4%	26.9%	0.0%	0.0%	73.1%
S7	2	5	4.31	0.805	50.0%	32.7%	15.4%	1.9%	0.0%	82.7%
S8	3	5	4.52	0.641	59.6%	32.7%	7.7%	0.0%	0.0%	92.3%

**Table 17:** Statistics of the scale of “user experience” questionnaire data.

Item No.	Min.	Max.	Mean	S. D.	Strongly Agree	Agree	Neutral	Disagree	Strongly Disagree	Agree or Above
T1	3	5	4.37	0.715	50.0%	36.5%	13.5%	0.0%	0.0%	86.5%
T2	3	5	4.29	0.776	48.1%	32.7%	19.2%	0.0%	0.0%	80.8%
T3	3	5	4.06	0.802	34.6%	36.5%	28.8%	0.0%	0.0%	71.1%
T4	3	5	4.35	0.711	48.1%	38.5%	13.5%	0.0%	0.0%	86.6%
T5	3	5	4.21	0.800	44.2%	32.7%	23.1%	0.0%	0.0%	76.9%
T6	3	5	4.19	0.841	46.2%	26.9%	26.9%	0.0%	0.0%	73.1%
T7	3	5	4.00	0.792	30.8%	38.5%	30.8%	0.0%	0.0%	69.3%
T8	3	5	4.37	0.658	46.2%	44.2%	9.6%	0.0%	0.0%	90.4%

**Table 18:** The measured values of the KMO test and the significance values of Bartlett’s test of the collected questionnaire data of the three scales as listed in Table 15, Table 16 and Table 17.

Scale	Name of Measure or Test		Value
User friendliness	KMO measure of sampling adequacy		0.792
	Bartlett test of sphericity	Approx. chi-square	171.686
		Degree of freedom	28
		Significance	0.000
User satisfaction	KMO measure of sampling adequacy		0.869
	Bartlett test of sphericity	Approx. chi-square	223.881
		Degree of freedom	28
		Significance	0.000
User experience	KMO measure of sampling adequacy		0.905
	Bartlett test of sphericity	Approx. chi-square	286.210
		Degree of freedom	28
		Significance	0.000

**Table 19:** Rotated component matrix of the first scale, “user friendliness”.

Variables	Component	
	1	2
R1	**0.840**	0.246
R3	**0.814**	0.099
R6	**0.749**	0.325
R2	**0.707**	0.207
R7	0.108	**0.807**
R8	0.312	**0.775**
R5	0.423	**0.722**
R4	0.130	**0.691**
Extraction method: principal component analysis. Rotation method: varimax with Kaiser normalization.		
a. Rotation converged in 4 iterations.		

**Table 20:** Rotated component matrix of the second scale, “user satisfaction”.

Variables	Component	
	1	2
S1	**0.891**	0.181
S2	**0.822**	0.292
S7	**0.741**	0.201
S8	**0.625**	0.298
S4	0.197	**0.813**
S5	0.521	**0.798**
S6	0.475	**0.717**
S3	0.517	**0.590**
Extraction method: principal component analysis. Rotation method: varimax with Kaiser normalization.		
a. Rotation converged in 3 iterations.		

**Table 21:** Rotated component matrix of the third scale, “user experience”.

Variables	Component	
	1	2
T2	**0.888**	0.273
T8	**0.743**	0.379
T1	**0.794**	0.424
T3	**0.636**	0.560
T4	**0.616**	0.538
T6	0.294	**0.746**
T5	0.322	**0.797**
T7	0.373	**0.527**
Extraction method: principal axis factoring. Rotation method: varimax with Kaiser normalization.		
a. Rotation converged in 3 iterations.		

**Table 22:** Collection of questions of the six latent dimensions of the three scales.

Scale	Title of Latent Dimension	No. of Questions	Labels of the Questions of the Dimension
User friendliness	Intention to act (Group RC1)	4	(R1, R3, R6, R2)
	Usefulness (Group RC2)	4	(R7, R8, R5, R4)
User satisfaction	Pleasure and relaxation (Group SC1)	5	(S7, S8, S2, S1)
	Cognitive stimulation (Group SC2)	5	(S4, S6, S3, S5)
User experience	Experience satisfaction (Group TC1)	4	(T2, T1, T8, T3, T4)
	Enrichment in life (Group TC2)	4	(T5, T6, T7)

**Table 23:** The questions of the three scales and the six latent dimensions, as well as corresponding Cronbach’s α coefficients.

Scale	Latent Dimension	Cronbach’s α Coefficient	Cronbach’s α Coefficient
User friendliness	Intention to act (Group RC1)	0.826	0.850
	Usefulness (Group RC2)	0.786	
User satisfaction	Pleasure and relaxation (Group SC1)	0.873	0.897
	Cognitive stimulation (Group SC2)	0.820	
User experience	Experience satisfaction (Group TC1)	0.926	0.924
	Enrichment in life (Group TC2)	0.802	

**Table 24:** Fitness indexes of the structural models of the two indicators of GPIC and the elderly’s attitude generated through CFA.

Scale	df	χ^2^	χ^2^/df	cfi	RMSEA	RMSEA (90% CI)	
						LO	HI
User friendliness	19	26.176	1.378	0.952	0.083	0.000	0.154
User satisfaction	19	30.779	1.620	0.942	0.106	0.020	0.172
User experience	19	22.018	1.159	0.989	0.054	0.000	0.135

**Table 25:** The construct validity values of the latent dimension of the three scales “user friendliness”, “user satisfaction”, and “user experience” generated through CFA.

Scale	Latent Dimension	Group of Related Questions	Construct Validity
User friendliness	Intention to act (Group RA)	RA = (R1, R3, R6, R2)	0.829
	Usefulness (Group RB)	RB = (R7, R8, R5, R4)	0.801
User satisfaction	Pleasure and relaxation (Group SA)	SA = (S7, S8, S2, S1)	0.872
	Cognitive stimulation (Group SB)	SB = (S4, S6, S3, S5)	0.819
User experience	Experience satisfaction (Group TA)	TA = (T2, T1, T8, T3, T4)	0.929
	Enrichment in life (Group TB)	TB = (T5, T6, T7)	0.817

**Table 26:** Analysis of responses to questions on the latent dimension “intention to act”.

Question		Min. Value	Max. Value	Mean	S. D.	Strongly Agree (5)	Agree (4)	Average (3)	Disagree (2)	Strongly Disagree (1)	Agree or Above
R1	I am more willing to try technology through eye interaction.	3	5	4.12	0.732	32.7%	46.2%	21.2%	0.0%	0.0%	78.9%
R3	In my leisure time, I think I will use this system frequently.	2	5	3.77	0.757	17.3%	44.2%	36.5%	1.9%	0.0%	61.5%
R6	I believe that the system interacting through eyes can effectively meet functional requirements.	2	5	4.10	0.721	28.8%	53.8%	15.4%	1.9%	0.0%	82.6%
R2	I believe that eye interaction can capture my interest.	3	5	3.98	0.700	23.1%	51.9%	25.0%	0.0%	0.0%	75.0%

**Table 27:** Analysis of responses to questions on the latent dimension “usefulness”.

Question		Min. Value	Max. Value	Mean	S. D.	Strongly Agree (5)	Agree (4)	Average (3)	Disagree (2)	Strongly Disagree (1)	Agree or Above
R7	I find eye interaction very easy.	3	5	4.27	0.660	38.5%	50.0%	11.5%	0.0%	0.0%	88.5%
R8	I am very confident in using the system.	3	5	4.21	0.723	38.5%	44.2%	17.3%	0.0%	0.0%	82.7%
R5	I think using eye interaction makes the system more convenient.	2	5	4.21	0.720	38.5%	46.2%	13.5%	1.9%	0.0%	84.7%
R4	I feel that interacting through eyes helps alleviate physical burden.	2	5	4.04	0.928	38.5%	32.7%	23.1%	5.8%	0.0%	71.2%

**Table 28:** Analysis of responses to questions on the latent dimension “pleasure and relaxation”.

Question		Min. Value	Max. Value	Mean	S. D.	Strongly Agree (5)	Agree (4)	Average (3)	Disagree (2)	Strongly Disagree (1)	Agree or Above
S7	I feel very relaxed during the experience process.	2	5	4.31	0.805	50.0%	32.7%	15.4%	1.9%	0.0%	82.7%
S8	I find the experience content interesting.	3	5	4.52	0.641	59.6%	32.7%	7.7%	0.0%	0.0%	92.3%
S2	I find the natural scenery presented in the visuals very beautiful.	3	5	4.38	0.718	51.9%	34.6%	13.5%	0.0%	0.0%	86.5%
S1	I really like the natural scenery presented in the visuals.	3	5	4.37	0.658	46.2%	44.2%	9.6%	0.0%	0.0%	90.4%

**Table 29:** Analysis of responses to questions on the latent dimension “cognitive stimulation”.

Question		Min. Value	Max. Value	Mean	S. D.	Strongly Agree (5)	Agree (4)	Average (3)	Disagree (2)	Strongly Disagree (1)	Agree or Above
S4	I can easily immerse myself in the experience process.	2	5	3.75	0.813	21.2%	34.6%	42.3%	1.9%	0.0%	55.8%
S6	After the experience, I feel more energetic.	3	5	4.06	0.777	32.7%	40.4%	26.9%	0.0%	0.0%	73.1%
S3	The experience process leaves a deep impression on me.	2	5	4.08	0.813	34.6%	40.4%	23.1%	1.9%	0.0%	75.0%
S5	The interactive feedback makes me curious.	3	5	4.31	0.701	44.2%	42.3%	13.5%	0.0%	0.0%	86.5%

**Table 30:** Analysis of responses to questions on the latent dimension “experience satisfaction”.

Question		Min. Value	Max. Value	Mean	S. D.	Strongly Agree (5)	Agree (4)	Average (3)	Disagree (2)	Strongly Disagree (1)	Agree or Above
T2	I feel that this experience activity helps me relax.	3	5	4.29	0.776	48.1%	32.7%	19.2%	0.0%	0.0%	80.8%
T1	This experience activity is very interesting to me.	3	5	4.37	0.715	50.0%	36.5%	13.5%	0.0%	0.0%	86.5%
T8	After the experience ends, I think it helps to increase willingness to participate in activities.	3	5	4.37	0.658	46.2%	44.2%	9.6%	0.0%	0.0%	90.4%
T3	I feel that this experience activity helps me relieve stress.	3	5	4.06	0.802	34.6%	36.5%	28.8%	0.0%	0.0%	71.1%
T4	I feel that this is a meaningful experience activity.	3	5	4.35	0.711	48.1%	38.5%	13.5%	0.0%	0.0%	86.6%

**Table 31:** Analysis of responses to questions on the latent dimension “enrichment in life”.

Question		Min. Value	Max. Value	Mean	S. D.	Strongly Agree (5)	Agree (4)	Average (3)	Disagree (2)	Strongly Disagree (1)	Agree or Above
T5	This experience activity enriches my daily life.	3	5	4.21	0.800	44.2%	32.7%	23.1%	0.0%	0.0%	76.9%
T6	I have a better understanding of the use of technology after the system experience.	3	5	4.19	0.841	46.2%	26.9%	26.9%	0.0%	0.0%	73.1%
T7	After the experience ends, I feel more active in interacting with others.	3	5	4.00	0.792	30.8%	38.5%	30.8%	0.0%	0.0%	69.3%

## Data Availability

The datasets used and/or analyzed during the current study are available from the corresponding author on reasonable request.
